# Multi-task benchmarking of spatially resolved gene expression simulation models

**DOI:** 10.1186/s13059-025-03505-w

**Published:** 2025-03-17

**Authors:** Xiaoqi Liang, Marni Torkel, Yue Cao, Jean Yee Hwa Yang

**Affiliations:** 1 School of Mathematics and Statistics, The University of Sydney, Sydney, NSW 2006 Australia; 2 Sydney Precision Data Science Centre, The University of Sydney, Sydney, NSW 2006 Australia; 3 Charles Perkins Centre, The University of Sydney, Sydney, NSW 2006 Australia; 4 Laboratory of Data Discovery for Health Limited (D24H), Science Park, Hong Kong SAR, China

## Abstract

**Background:**

Computational methods for spatially resolved transcriptomics (SRT) are often developed and assessed using simulated data. The effectiveness of these evaluations relies on the ability of simulation methods to accurately reflect experimental data. However, a systematic evaluation framework for spatial simulators is currently lacking.

**Results:**

Here, we present SpatialSimBench, a comprehensive evaluation framework that assesses 13 simulation methods using ten distinct STR datasets. We introduce simAdaptor, a tool that extends single-cell simulators by incorporating spatial variables, enabling them to simulate spatial data. SimAdaptor ensures SpatialSimBench is backwards compatible, facilitating direct comparisons between spatially aware simulators and existing non-spatial single-cell simulators through the adaption. Using SpatialSimBench, we demonstrate the feasibility of leveraging existing single-cell simulators for SRT data and highlight performance differences among methods. Additionally, we evaluate the simulation methods based on a total of 35 metrics across data property estimation, various downstream analyses, and scalability. In total, we generated 4550 results from 13 simulation methods, ten spatial datasets, and 35 metrics.

**Conclusions:**

Our findings reveal that model estimation can be influenced by distribution assumptions and dataset characteristics. In summary, our evaluation framework provides guidelines for selecting appropriate methods for specific scenarios and informs future method development.

**Supplementary Information:**

The online version contains supplementary material available at 10.1186/s13059-025-03505-w.

## Background

Spatial transcriptomics (ST) technology represents a significant advancement in the field of molecular biology, offering the ability to map gene expression data within the spatial context of tissue samples [1]. In many situations, the ground truth, such as differential expression or differential gene abundance, is experimentally unattainable. Simulation, in contrast, provides access to a controlled environment with ground truth, thereby enabling systematic evaluation of algorithms. Spatial simulations play an essential role in validating the efficacy of computational tools such as CARD [2], stLearn [3], SPIAT [4], and BayesSpace [5]. These tools address a range of challenges, including cell type deconvolution, cell–cell interaction analysis, tissue microenvironment characterization, and sub-spot resolution enrichment. Hence, computational simulation methods that generate spatial datasets stand as a crucial strategy for assessing the performance of spatial analytical tools.

In a recent study, simBench [6] has recognized the importance of simulated datasets for methodology development and provides a platform for assessing how well various simulation tools reflect real-world data. With the increased demand for spatial analytical tools, there is an emerging but limited number of spatially aware simulators developed to assist with method development. We can categorize spatially aware simulators based on the type of input: spot-level data (as in Visium ST) and scRNA-seq data. Spot-level data simulators, such as scDesign3 [7] and SRTsim [8], generate spot-level count data while preserving the spatial layout observed in real data. scDesign3 focuses on reference-based simulations, whereas SRTsim can handle both reference-based and reference-free scenarios.

Another category utilizes scRNA-seq data as input. Simulators like spider [9], stLearn [3], and SpatialcoGCN-sim [10] fall into this category, generating spot-level count data and spatial location. While some scRNA-seq based simulators, such as spaSim [4], focus solely on cell location simulation, others, like scMultiSim [11] and stLearn [3], can simulate both cell counts and spatial cell–cell interaction relationships. It is important to note that some simulators used in publications, such as CARD [2], are not published independently but rather used as part of the evaluation process for published methods.

Considering the large number of single-cell simulators currently available and the relatively few spatially aware simulators that accept spot-level data as input, it is essential not to overlook existing tools or to start from scratch when developing spatial simulators. One might investigate the opportunities to adapt the capabilities of current single-cell simulators. These single-cell simulators are highlighted in the simBench [6] study, which includes SPARsim [12], ZINB-WaVE [13], Splatter [14], and SymSim [15].

To this end, our study, SpatialSimBench, is the first single-cell benchmarking study to examine simulation approaches with specific attention to spatial expression. In particular, we address the opportunity of leveraging existing single-cell simulators and have developed simAdaptor, a tool that enables the extension of single-cell simulators to simulate spatial data by incorporating spatial variables. We have devised different simulation strategies to make the benchmarking “backwards” compatible. That is, we examine in one frame between (i) spatially aware simulation models and (ii) existing “non-spatial” simulation methods that are adapted. Our benchmarking design will leverage and extend the framework developed in our previous work, simBench. It will (i) examine both two input types of spatially aware simulators methods; (ii) introduce spatially specific metrics to examine data properties (simBench); and (iii) examine impact on multiple downstream analysis tasks that are typically done in spatially analysis. Finally, we compile the findings into recommendations for users and emphasize potential areas for future research.

## Results

### SpatialSimBench is a comprehensive benchmark of spatially resolved simulation methods using diverse datasets and comparison measures

**Fig. 1:**
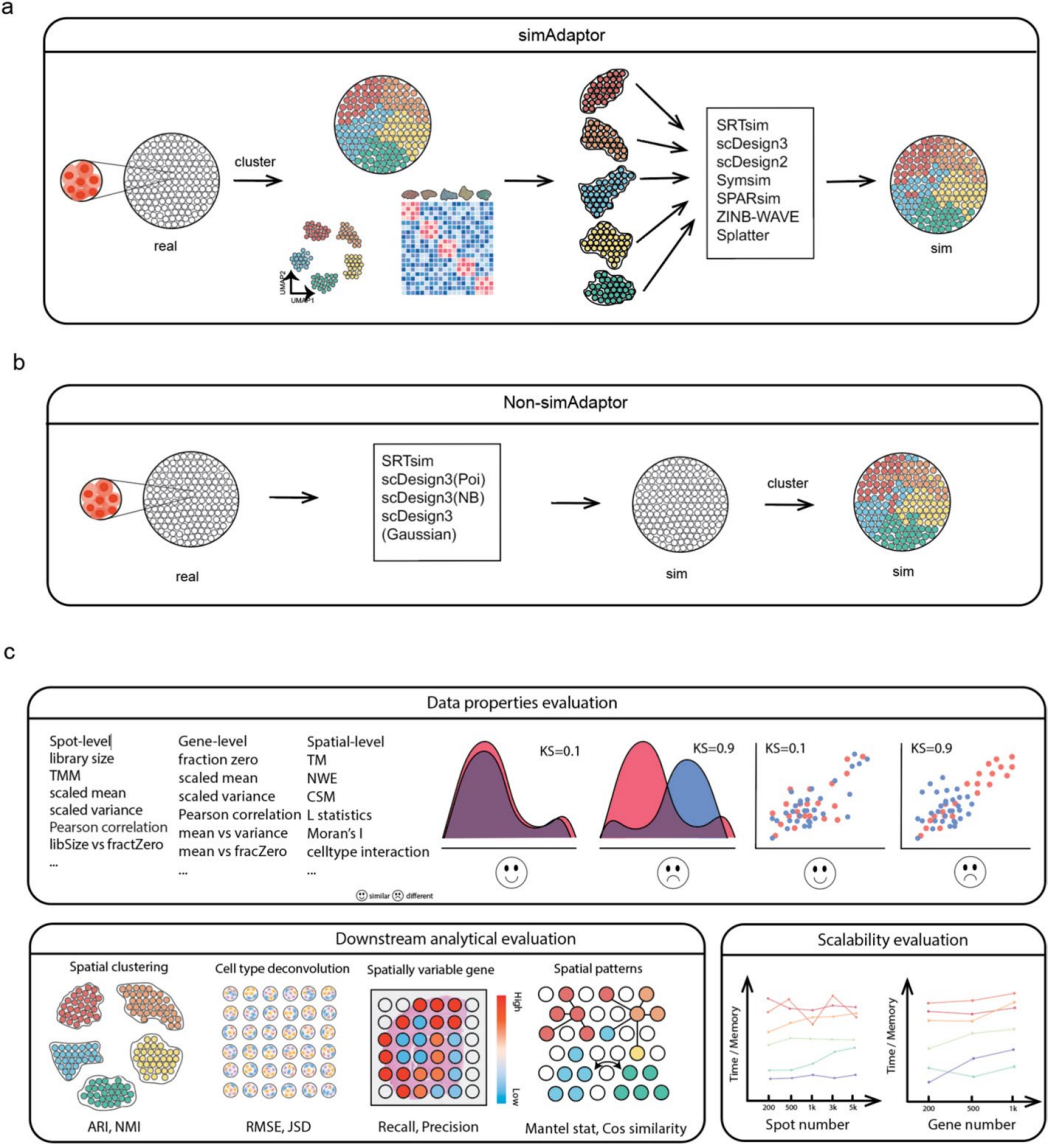
Overview of the benchmarking process and key aspects of evaluation. **a** Schematic of simAdaptor approach. It applies spatial simulated models directly without segmentation considerations. **b** Schematic of non-simAdaptor approach. Initially, spatial clustering of data identifies regions sharing similar expression profiles. Following this, spatial transcriptomics data is categorized, allowing the application of established simulation methods to each identified category. **c** Multi-tasks of evaluation were examined in this study, including data properties, spatial downstream analytical task, and scalability

Our SpatialSimBench framework evaluates recently published spatially aware simulation methods together with single-cell simulation methods adapted with simAdaptor (Fig. 1a, Additional file 1: Table S1) and without simAdaptor for comparison (Fig. 1b). The simAdaptor (see more details in the next results section) is used to leverage the large collection of existing single-cell simulators for spatial simulation by incorporating spatial variables into single-cell simulators. This SpatialSimBench includes a total of 35 metrics that comprehensively examine data property estimation (spot-level, gene-level, and spatial-level) and a diverse range of spatial tasks (spatial clustering, spatially variable gene identification, cell type deconvolution, and spatial cross-correlation), as well as scalability. In our previous study, we introduced simBench, a benchmark study of simulation methods for scRNA-seq data. We leverage the various categories of gene and cell level properties and scalability developed in simBench and introduce three additional categories of spatial-specific metrics. The first category refers to the simulator’s ability to capture data properties of spot-level and spatial-level and the second focuses on the simulator’s performance capacity in various spatial downstream tasks. The third category is scalability, including time and memory measurement at different scales. In the three categories, we used the smiley face to denote cases where the method exhibits robust performance. The frowny face indicates areas where the method demonstrates limited performance, suggesting potential room for improvement. Similar to simBench, to ensure robustness and generalizability of the study results and account for variability across datasets, we collect ten public spatial transcriptomics experimental datasets, encompassing a variety of sequencing protocols, tissue types, and health conditions from human and mouse sources. Spatial simulation data was generated by using these real experimental datasets as a reference. The simulation was then assessed against real data using the three metric categories (Fig. 1c). Through this systematic comparison, we generated a total of 4550 results derived from ten spatial datasets, 13 simulation methods, and 35 metrics.

To assess the similarity between real dataset and simulated dataset in data properties, we use both cell-level (spot-level in spatial data) and gene-level metrics. This involves defining metrics encompassing various aspects, including spot density, along with higher-order interactions, such as mean–variance relationships. To capture the spatial dimension, we evaluate how well each simulator captures spot-spot relationships in the spatial setting, which is referred to as the spatial-level in the data properties section. This is achieved by analyzing both real and simulated data with transition matrices, neighborhood enrichment matrices, and centralized score matrices to quantify spatial relationships between spots (see Methods). Also, we added spatial metrics (cell type interaction, *L* statistics, nn correlation, and Moran’s *I*) from our previous study called scFeatures [16], multi-view representations of single-cell and spatial data at the spot-level. We also propose spatially aware metrics to evaluate a simulation model’s ability to capture spatial patterns, cell type composition, spatially variable gene identification, and spatial cross-correlation of real data. To evaluate the similarity between simulated and real data for each metric, we utilized two methods: (1) density plots for visual inspection and (2) the kernel density-based global two-sample comparison (KDE) test statistic [17] for quantitative assessment. A lower KDE test statistic indicates a smaller difference between the real and simulated data distribution (Additional file 1: Figs. S1 and S2) which indicates a better performance. To assess the efficiency of our simulation models in generating large-scale datasets, we investigated computational scalability. This involved measuring the simulation models’ running time and memory usage while simulating datasets with varying numbers of spots and genes.

To examine how well the simulator captures some of the standard downstream analysis, we focus on downstream tasks. These tasks include (1) maintaining spatial clustering, measured by Adjusted Rand Index (ARI) and Normalized Mutual Information (NMI); (2) performing cell-type deconvolution, where simulated data are compared to real data using the same deconvolution algorithm and evaluated using Root Mean Square Error (RMSE) and Jensen-Shannon Divergence (JSD); (3) accurately identifying spatially variable genes (SVG), comparing simulated data to real data using the same detection method and measuring by recall and precision; and (4) evaluating spatial cross-correlation (bivariate Moran’s *I*), and evaluated using Mantel statistics and cosine similarity to assess how well the simulation reflects the actual spatial cross-correlation.

### Leverage existing scRNA-seq simulation for spatial resolved data to capture spatial patterns

To examine whether we can leverage the extensive collection of existing single-cell simulations for spatial simulation, we developed the simAdaptor method that incorporates spatial variables into single-cell simulators. The strategy begins by employing spatial clustering to identify groups of regions with similar gene expression profiles. This approach relies on the assumption that distinct spatial clusters will harbor transcriptional features within their respective regions. In the initial step, clusters are manually created with the assumption that each cluster represents a distinct spatial region. Subsequently, each cluster is then utilized as input for spatial or single-cell simulation models to simulate individual spatial regions. We term this approach simAdaptor, which uses regional information as the foundation.

**Fig. 2:**
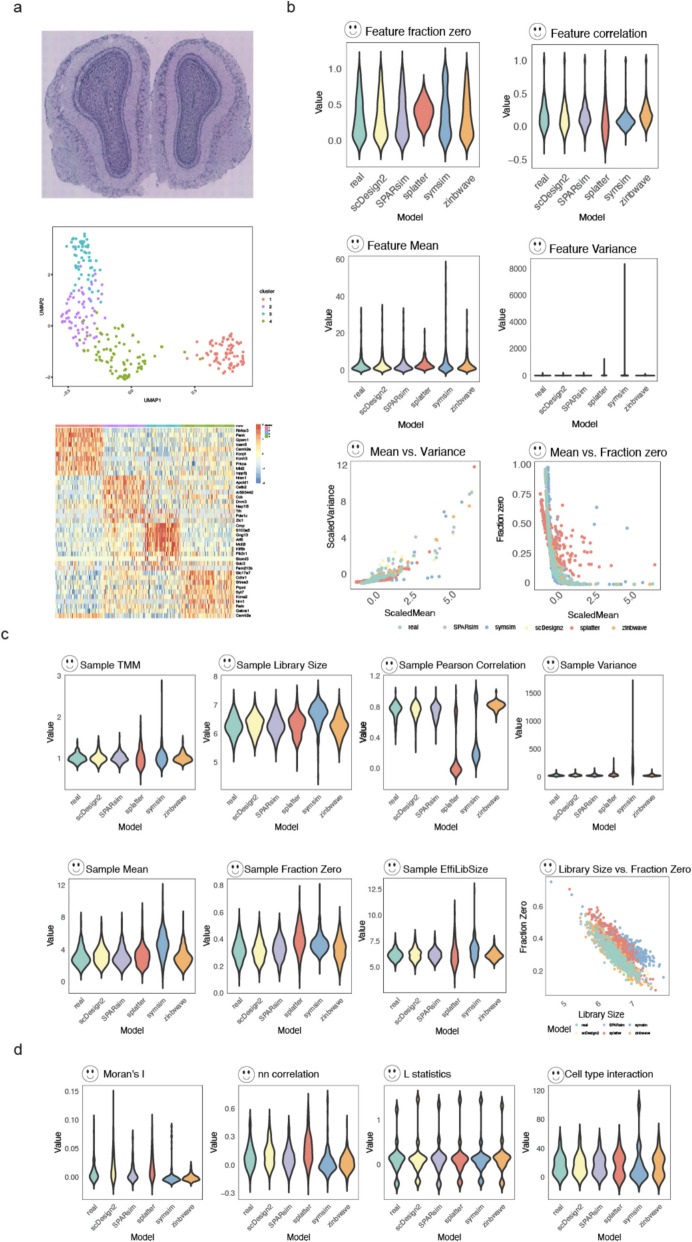
Overview of the simAdaptor approach with data properties evaluation result. **a** Performance of spatial clustering and differential expression analysis on Dataset 8. **b** Visualize the real and simulation in boxplot across gene-level metrics. **c** Visualize the real and simulation in boxplot across spot-level metrics. **d** Visualize the real and simulation in boxplot across spatial-level metrics

**Fig. 3:**
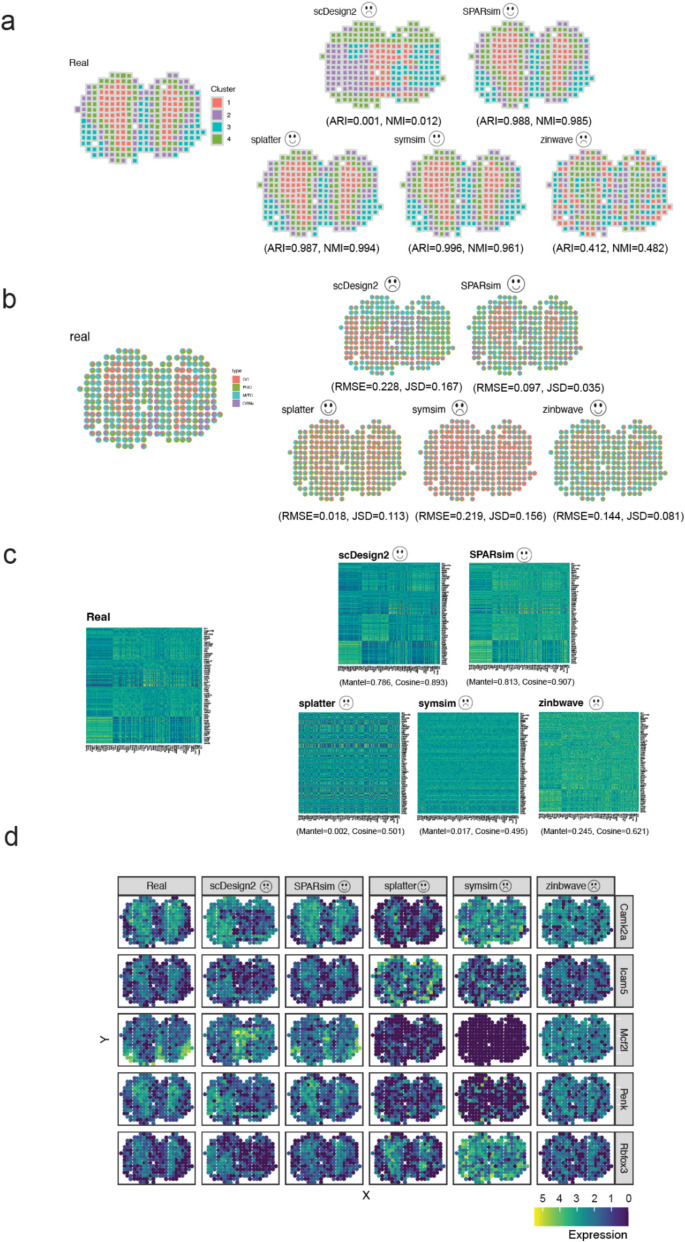
Overview of the simAdaptor approach with spatial downstream analysis result. **a** Spatial clustering visualization comparison. **b** Cell type deconvolution visualization comparison. **c** Spatial cross correlation visualization comparison. **d** Selected spatial variance gene visualization comparison

To illustrate the efficacy of the simAdaptor, we generated spatial simulation data using adult mouse olfactory bulb spatial gene expression data as reference (Fig. 2). Figure 2a demonstrates the initial spatial clustering of the data into four distinct groups. Differential gene expression analysis is conducted within individual spatial clustering groups to highlight distinct gene expression patterns associated with each group. Following this, five single-cell simulators were employed to assess their ability to capture data distribution of the spatial data. From Fig. 2b and c, the simulation data from scDesign2, SPARsim, and ZINB-WaVE show similar distribution compared with real data particularly in gene-level and spot-level. In spatial-level evaluation, scDesign2, SPARsim, and Splatter outperform others (Fig. 2d). In evaluating the performance on the adapted simulator of identifying regional structure, we found the adapted methods based on SPARsim, Splatter, and SymSim show consistent spatial clustering patterns with real data (Fig. 3a). Similarly, SPARsim, Splatter, and ZINB-WaVE effectively capture the majority of cell type proportions observed in the real data (Fig. 3b). In spatial autocorrelation, scDesign2 and SPARsim outperformed (Fig. 3c). SPARsim and Splatter performed well in selected spatially variable genes (Fig. 3d). Our analysis of spatial patterns revealed that specific single-cell simulators, including SPARsim, Splatter, ZINB-WaVE, and SymSim, generated spatial patterns consistent with those observed in the real spatial data. Additionally, spot-spot relationships, such as cell-type interactions and Moran’s *I*, exhibited similar distributions to the real data. These findings demonstrate the effectiveness of our approach in adapting single-cell simulators for generating data with spatial features.

Next, we assess the applicability of the simAdaptor approach to four spatially aware simulators and whether this approach improves their performance (Additional file 1: Fig. S3). Visualizations across the spot-level, gene-level, and spatial-level metrics revealed higher degree of similarity between the simAdaptor and non-simAdaptor, suggesting compatibility of simAdaptor approach with both spatial and single-cell simulators. Notably, our findings indicate that the simAdaptor approach led to improvements in specific performance metrics for spatial simulators, particularly in spot-wise scaled mean and variance.

### Relative performance on data properties and scalability evaluation criteria

**Fig. 4:**
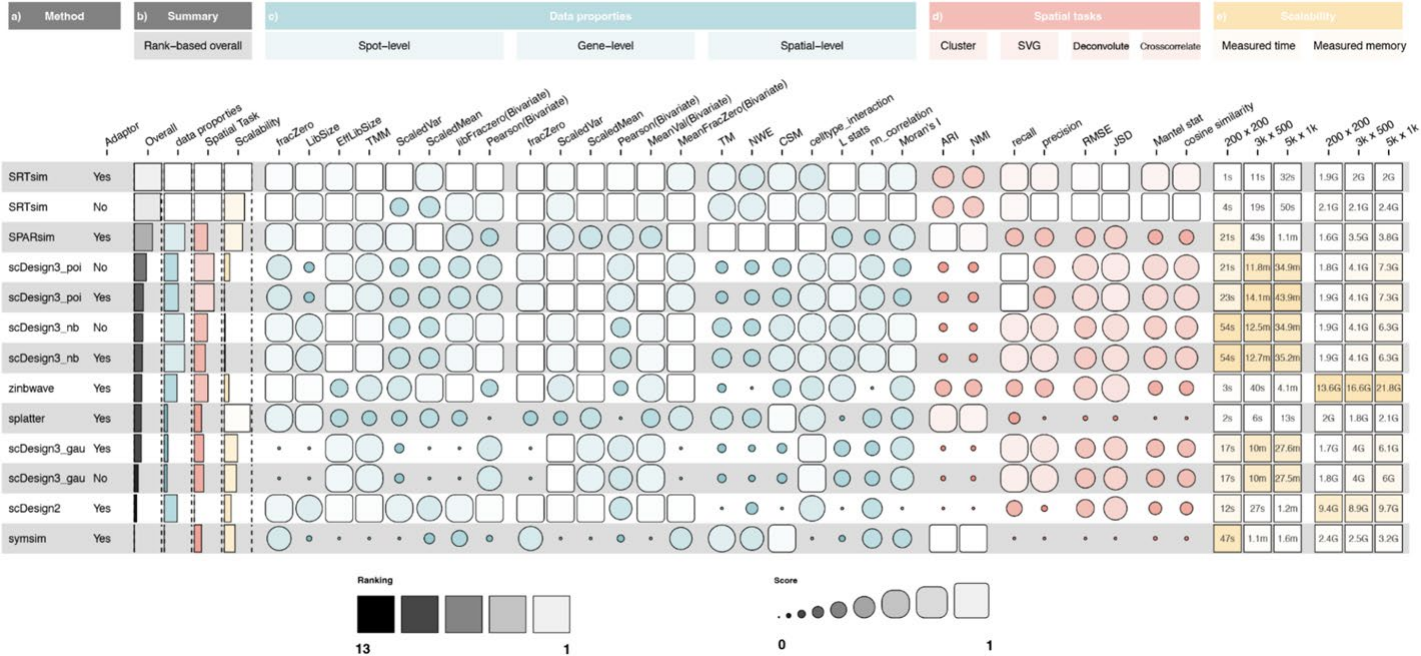
Details result of three main evaluation metrics: data properties, spatial task, and scalability. The color represents different areas of evaluation and the higher score shows the best possible rank of 1. **a** The name of the method across non-simAdaptor and simAdaptor approaches. **b** Summary of all the overall performance. We used different distribution parameters of scDesigns, where poi is Poisson distribution, nb is negative binomial, and gau is Gaussian distribution. **c** Score of methods within data properties, ranking by KDE test statistics. **d** Score of methods within spatial tasks, ranking by specific metrics. **e** Scalability results for varying numbers of spots and features (number of spots × number of features). K, thousands

Using simAdaptor, we evaluated a total of 13 simulation methods, including five single-cell simulators and eight spatial simulators. Analysis of these 13 simulation methods (Fig. 4a) across data properties and scalability evaluations (Fig. 4c, e) revealed variable method performance across metrics. As expected, the performance of the method varied significantly among evaluation metrics, suggesting that there is not a universally effective approach that performs well across data properties and scalability evaluation. This section will first examine data properties estimation including gene-level, spot-level, and spatial-level. After that, we will evaluate scalability including measured time and memory with varying numbers of genes and spots, to determine the computational efficiency and feasibility of the simulators for large-scale datasets.

Interestingly, we observed that certain single-cell simulators such as scDesign2, ZINB-WaVE, and SPARsim are equally as good as spatial simulators such as scDesign3 and SRTsim in capturing gene-level and spot-level data distributions. Nevertheless, the spatial simulator SRTsim is the only method that consistently excelled in spatial metrics. This is not unexpected, given that spatial simulators are specifically designed to capture spatial relationships. In detail, for gene-level estimation, the performance of methods varied across six criteria. Among these, the single-cell methods scDesign2, ZINB-WaVE, and SPARsim and the spatial methods SRTsim and scDesign3 (poi) outperformed the others (Fig. 4c). For the other methods, significant differences were noted across the six criteria, with no clear patterns or correlations in their rankings based on each criterion. For spot-level estimation, we also noticed variability in the performance of methods across eight criteria. The single-cell methods scDesign2, ZINB-WaVE, and SPARsim and the spatial methods SRTsim and scDesign3 (nb) emerged as top performers (Fig. 4c). It is important to note that SRTsim with the simAdaptor yielded better results than without simAdaptor when assessing scaled variance and mean at the spot-level. For spatial-level estimation, we found that some single-cell simulators, such as SPARsim and Splatter, perform well. They are as effective as certain spatial simulators, such as SRTsim and scDesign3.

Evaluating computational efficiency, each method was tested on subsampled versions of a real dataset (Fig. 4e, Additional file 1: Fig. S4). Most exhibited good performance, with runtimes under one hour and memory consumption below ten gigabytes (GB). Notably, most single-cell simulators generally excelled in both aspects. However, a trade-off between efficiency and modeling complexity emerged: ZINB-WaVE achieved top running speeds at the cost of high memory requirements, while scDesign2 demonstrated efficient memory usage but had longer runtimes compared to scDesign3. scDesign3 utilizes a copula model to capture correlation, which can be time-consuming, especially as the number of genes scales. In this work, we need to constrain the number of genes (200, 500, 1k) in the dataset to ensure the simulation could be completed within reasonable timeframes. This also highlights the inherent tension between computational demands and the sophistication of the simulation framework. No significant distinction was observed between spatially aware and simAdaptor approaches in terms of scalability.

### Downstream analytical tasks revealed their relative performance on multi-tasks criteria

Next, we examined spatial tasks such as spatial clustering, spatially variable gene identification, cell type deconvolution, and spatial cross-correlation (Fig. 4d). The objective was to determine the realism of simulated data in subsequent analyses. We observed variability in methods’ performance for four spatial tasks. This also suggests that no single method consistently excels in all spatial task evaluations. In general, most single-cell simulations and spatial simulators performed well in the four spatial tasks evaluated (Fig. 4d). Our evaluation shows that the single-cell methods SPARsim, Splatter, and SymSim as well as the spatial method SRTsim excel in spatial clustering performance. In the spatial task of cell type deconvolution, we observed that the spatial methods SRTsim and scDesign3 generally outperformed single-cell simulators. For spatially variable gene identification, the single-cell method ZINB-WaVE, along with the spatial methods SRTsim and scDesign3, significantly outperformed others. Additionally, unlike spatial autocorrelation (univariate Moran’s *I*), which assesses how a single variable correlates with itself across different spatial locations, spatial cross-correlation explores how two distinct variables co-vary spatially. Our analysis demonstrated that the spatial methods SRTsim and scDesign3 (poi) significantly outperformed other simulators in this task.

To strengthen the robustness of SpatialSimBench, we assessed the concordance in downstream method rankings between real and simulated datasets. This approach is based on the rationale that effective simulation data should produce a similar ranking of downstream methods to that observed with real data. Specifically, we applied five SVG identification methods (SPARK-X [18], nnSVG [19], MERINGUE [20], Seurat’s HVG [21], and Giotto [22]) and six spatial clustering methods (BayesSpace [5], Seurat’s Leiden [21], PRECAST [23], DR.SC [24], BASS [25], and SpatialPCA [26]). The details of these tools are in Additional file 1: Table S2 and Additional file 1: Table S3. The concordance index is calculated between the evaluation results between simulated and real datasets (Additional file 1: Fig. S5). Results indicated that SRTsim, SPARsim, and Splatter had higher concordance indices, suggesting that they more accurately mirrored the performance hierarchy of tools observed in real data. Moreover, there is a high level of concordance in the second layer, with an overall correlation of 0.78, which further supports the consistency of our results as previously demonstrated.

### Impact of model distribution and dataset characteristic on model performance

Beyond comparing overall method performance, understanding the factors influencing simulation outcomes is crucial for informed method selection and method development. Here, we investigate the potential factors influencing simulation results, identifying both the common strengths and weaknesses of current simulation methods, as well as the progress achieved. We first explored the influence of distribution assumptions on model estimates by applying different distributions (Gaussian, negative binomial, Poisson) to scDesign3. Negative binomial performed the best, followed by Poisson and Gaussian distributions. This observation corroborates the typical distribution modeling approach in the single-cell community, where data are commonly modeled as negative binomial or Poisson distribution rather than Gaussian distribution. This suggests that capturing overdispersion, a common feature of spatial data, is crucial for accurate modeling. While SPARsim and ZINB-WaVE (utilizing zero-inflated negative binomial and gamma distributions, respectively) maintained good performance, SRTsim excelled across most metrics. This potentially stems from their ability to adaptively select distributions (Poisson, negative binomial, zero-inflated Poisson, and zero-inflated negative binomial) based on the data, offering greater flexibility in capturing complexities.

**Fig. 5:**
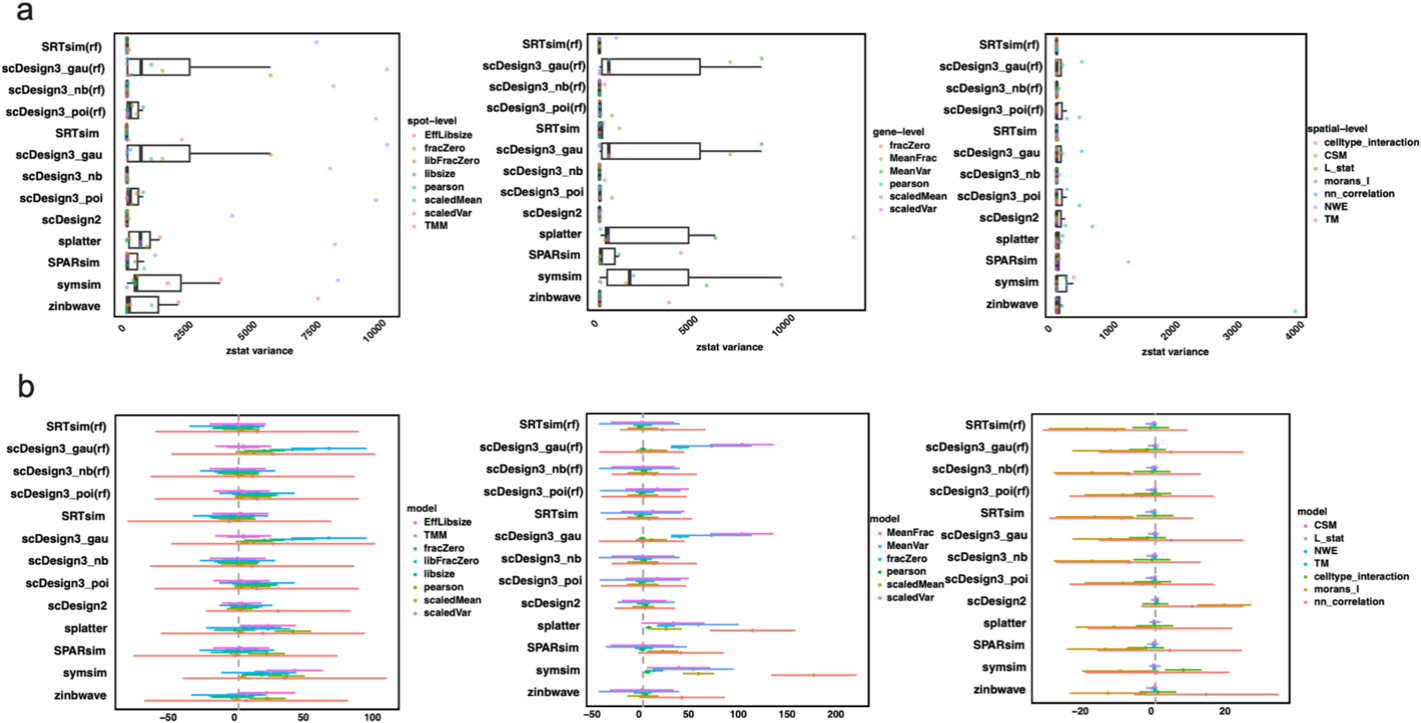
Impact of dataset and evaluation metric on method performance. **a** Dataset influence on models, illustrated by a boxplot depicting model-dataset consistency in spot-level, gene-level, and spatial-level; **b** Forest plot represents the impact of submetrics across different models. There are three graphs in each panel representing different evaluation areas, starting from left to right: spot-level, gene-level, and spatial-level

To examine if the performance of simulation models is consistent across datasets, we examined the KDE test statistics values across various data properties (spot-level, gene-level, and spatial-level) on different datasets (Fig. 5). The scDesign3 (nb), SRTsim, and scDesign2 displayed superior consistency across spot-level, gene-level, and spatial-level evaluations (Fig. 5a). This is further supported by the forest plot analysis where SRTsim, scDesign3 (nb), and scDesign2 exhibited minimal variability across evaluation metrics. The scDesign3 (gau) was affected by fraction zero, library size, and Pearson correlation, potentially due to scDesign3’s Gaussian assumption being unsuitable for sparse data. The Splatter, SymSim, and ZINB-WaVE were significantly influenced by spot-spot Pearson correlation and efficient library size (Fig. 5b). In gene-level, we observed that scDesign3 (gau), Splatter, and SymSim’s performance was influenced by higher-order interactions such as mean versus variance and mean versus fraction zero. Most of the single-cell simulators (Splatter, SPARsim, SymSim, and ZINB-WaVE) were affected by scaled variance and scaled mean. In spatial-level, we found that most simulators are strongly influenced by how a single variable correlates with itself across different locations. This relationship is measured by Moran’s *I*. These findings highlight the importance of considering the data type when selecting models and the implementation of a comprehensive collection of data types and evaluation metrics when assessing simulation models.

## Discussion

In this study, we present SpatialSimBench, a multi-task benchmarking study evaluating the performance of overall simulation methods in spatial gene expression data, including eight spatial simulators and five single-cell simulators. Importantly, we introduced a simulation strategy, which we termed simAdaptor. We demonstrated that simAdaptor enables existing scRNA-seq simulators to simulate spatially resolved data, as well as improving the performance of spatial simulators. We assessed all simulators using ten spatial gene expression datasets with paired single-cell gene expression data and analyzed them across 35 distinct metrics. These metrics cover a range of aspects, including data properties, spatial downstream analytical tasks, and scalability. Based on our results, we also explored the effect of distribution assumptions and the consistency of data characteristics on model estimation. Overall, this study provides recommendations for method selection and identifies improvements in future method development.

A major challenge for spatial simulators lies in the heterogeneity of spatial transcriptomics (ST) technologies. For example, image-based ST, such as seqFISH [27] and MERFISH [28], offers high resolution but is limited by a smaller number of target genes. Sequencing-based ST captures expressed RNAs in space but with low spatial resolution, such as ST [29] and 10X Visium [30]. One key finding was the significant impact of sparsity in the spatial transcriptomics data matrix on simulation performance. Methods like scDesign3 (gau) appear to have limited effectiveness on higher-order interactions. This limitation might be because scDesign3’s underlying assumption of Gaussian distribution may not be appropriate for data that is sparse or has many zeros. Moreover, our analysis further revealed that several single-cell simulators, including SPARsim, Splatter, and SymSim, exhibit robust capabilities for handling spatial clustering in downstream analysis. These tools are originally designed for managing complex scenarios inherent and they also retain these features when using simAdaptor.

We also explore the idea that current models for simulating single cells could be slightly modified to analyze spatial gene expression. We raise the question of whether a spatially aware simulator adds practical advantage or is it mainly a theoretical justification. In this study, we developed a simAdaptor approach that utilizes existing simulators to generate spatial gene expression data by incorporating spatial segmentation techniques. This approach successfully captured some aspects of real spatial data, including clustering patterns and similar spatial distribution patterns. Yet, when it comes to capturing complex interactions of features across spots and genes, specialized spatial simulators perform better. These spatial simulators prove useful in certain tests, but there is still a lot to gain from integrating single-cell simulation techniques. This raises the question of future method development. Should we modify existing single-cell simulators into spatial versions, or design entirely new structures specifically for spatial data? An interesting possibility lies in combining these approaches, potentially leading to new and powerful methods within the single-cell research community.

Our findings from both SpatialSimBench and simBench studies show remarkable consistency. Both sets of simulations observed that: (1) different methods perform differently depending on the criteria used for evaluation; (2) there is an inherent trade-off between computational efficiency and achieving a good model fit; and (3) the underlying distribution assumptions and datasets used can significantly impact model estimation. While some observations are similar between simBench and SpatialSimBench, there have been significant advancements in the past 4 years. Notably, simBench studies highlighted that most of the simulation models often underperform higher-order interaction metrics. Conversely, SpatialSimBench studies demonstrate substantial improvement in both spatial and single-cell simulators. More importantly, SpatialSimBench establishes a novel benchmarking framework that bridges the gap in single-cell RNA sequencing simulations by incorporating spatial context. This innovation emphasizes the potential for creative benchmarking approaches in the single-cell community, even with ongoing method development, as these methods can still be significantly influenced by the chosen evaluation framework. In essence, both studies confirm the importance of considering evaluation criteria, computational cost, and underlying assumptions when selecting methods. However, SpatialSimBench takes a significant leap forward by introducing a creative benchmarking strategy that integrates spatial context, paving the way for future advancements in the field.

Recently, the Open Problems in Single-Cell Analysis [31] offered an open-source, community-driven platform for benchmarking various formalized tasks in single-cell analysis. It covers a broad range of areas, including batch integration, cell–cell communication inference, and spatial decomposition contributed through community effort. In developing interactive software for SpatialSimBench, we aim to contribute to the broader single-cell analysis community through the Open Problems Framework and also to offer a more detailed and interactive experience by developing our own Shiny app, which can be accessed at https://sydneybiox.github.io/SpatialSimbench_website/results/.

## Conclusions

In summary, our work offers valuable insights for both benchmarking and method development in spatial transcriptomics. We demonstrated the usefulness of our framework by summarizing how different methods perform across various aspects. This can help users select the appropriate method for their needs while identifying areas where developers can improve existing methods. Significantly, we developed a simulation approach called simAdaptor, which adapts existing scRNA-seq simulators to generate spatially resolved data and enhances the performance of spatial simulators. Furthermore, we have introduced a novel benchmark evaluation framework in spatial simulation by integrating spatial information into traditional single-cell RNA sequencing simulations. Our findings are a valuable resource for both biologists seeking to analyze their spatial transcriptomics data and method developers aiming to advance the state-of-the-art.

## Methods

### Data description

In this benchmark study, we collected a total of ten spatial transcriptomics datasets alongside reference scRNA-seq datasets. The spatial transcriptomics datasets include a range of protocols, tissue types, and health conditions, from human and mouse. The scRNA-seq datasets were obtained by 10 × Chromium and Smart-seq platforms with all cell type labels being accessible publicly. Details of all datasets are available in Additional file 1: Table S4.

Regarding data preprocessing, we normalized the expression matrix for spatial transcriptomics datasets and scRNA-seq datasets using scater [32]. In consideration of time constraints and the convention in single-cell research that usually considers a selection of 1000 to 2000 genes adequate for downstream analysis, we selected the top 1000 spatially variable genes when the total number of genes exceeded 1000; otherwise, all genes were included. For the scRNA-seq datasets, our focus was on ensuring data integrity, by following a stringent quality control process [33]. Following the QC metric from Seurat [34], we filtered cells with unique feature counts over 2500 or less than 200 and more than 5% mitochondrial counts.

To estimate cell-type composition, we employed BayesSpace as it outperforms other cell type clustering methods for spatial transcriptomics data [35]. CARD integrated cell-type-specific information from scRNA-seq data with correlation in cell-type composition across tissue location, achieved through the implementation of a conditional autoregressive (CAR) modeling approach.

### simAdaptor

Our study introduces a novel approach that integrates spatial context into traditional single-cell RNA sequencing (scRNA-seq) simulation models. We termed this approach simAdaptor. This involves a two-step process. Initially, spatial data is clustered to identify regions with similar expression profiles. In this work, we used BayesSpace, a method designed specifically for spatial clustering. After clustering, we applied established simulation methods to simulate the data separately in each cluster. This process enables us to indirectly incorporate spatial variables into existing simulation models. In comparison, the non-simAdaptor approach applies spatial simulation onto each dataset directly without modification.

### Benchmark method

Our literature review identified six spatial simulators: scDesign3 [7], SRTsim [8], scMultiSim [11], stLearn [3], Spider [9], and spaSim [4]. While scMultiSim employs cell differentiation trees and gene regulatory networks to simulate spatial gene expression data at the cell level, this approach does not take gene expression as the input. It is thus not possible to assess whether this method performs well on gene-level, spot-level, and spatial-level data properties. On the other hand, Spider and stLearn process scRNA-seq data to simulate count data along with spatial locations. However, their generated spatial data are challenging to evaluate. Specifically, the absence of ground truth spatial location information in their outputs makes it difficult to compare with spatial patterns. SpaSim only stimulates cell locations by simulated image without spatial data. Therefore, we excluded these four spatial simulators for two primary reasons: the unsuitability of their input formats in capturing both spatial and gene expression details, and the inherent difficulties in comparing simulated spatial patterns without verifiable spatial location information. To ensure a focus on spatial data generation with a fair comparison, we selected scDesign3 and SRTsim. To compare how spatial simulators compare with existing single-cell simulators. We further expanded our analysis to include well-performing single-cell simulators from the SimBench study, such as Splat [14], ZINB-WaVE [13], SymSim [15], scDesign2 [36], and SPARsim [12]. All of the simulation models are implemented in R. Comprehensive information about these methods, including code versions, associated publications, and default parameter configurations, can be found in Additional file 1: Table S1.

### Evaluation of data properties

We developed a unified pipeline to assess the efficacy of the simulation models. Within this pipeline, the first evaluation is data properties including spot-level, gene-level, and spatial-level. The details of three evaluations categories with metric and range are available in Additional file 1: Table S5. After computing the metric for both the real and simulated datasets, we employ density plots and kernel density scores for each metric to assess the similarity between the real and simulated datasets.

### Spot-level

- Library size: total sum of UMI counts across all genes.
- TMM: trimmed mean of *M*-values normalization factor, where trimming is applied to both log-fold-changes and absolute intensities [37]
  $${\text{log}}_{2}\left({\text{TMM}}_{k}\right)=\frac{{\sum }_{g\in {G}^{*}}{w}_{gk}^{r}{M}_{gk}^{r}}{{\sum }_{g\in {G}^{*}}{w}_{gk}^{r}}$$
  $${\text{where}}\hspace{1em}{M}_{gk}^{r}=\frac{{\text{log}}_{2}\left(\frac{{Y}_{gk}}{{N}_{k}}\right)}{{\text{log}}_{2}\left(\frac{{Y}_{gr}}{{N}_{r}}\right)}\hspace{1em}{\text{and}}\hspace{1em}{w}_{gk}^{r}=\frac{{N}_{k}-{Y}_{gk}}{{N}_{k}{Y}_{gk}}+\frac{{N}_{r}-{Y}_{gr}}{{N}_{r}{Y}_{gr}};\hspace{1em}{Y}_{gk},{Y}_{gr}>0$$
  where ${Y}_{gk}$ is the observed count for gene $g$ in sample $k$ (test library), ${N}_{k}$ is the total number of reads in sample $k$, and ${G}^{*}$ is the set of not trimmed genes. For reference sample $r$, ${Y}_{gr}$ and ${N}_{r}$ represent the corresponding values. All counts are summarized from raw sequencing reads.
- Effective library size: library size multiplied by TMM.
- Scaled variance: *z*-score standardization of the variance of expression matrix in terms of log2 CPM
  $${z}_{ij}=\frac{{x}_{ij}-{\mu }_{j}}{{\sigma }_{j}}$$
  where ${x}_{ij}$ is the ${\text{log}}_{2}\text{CPM}$ value for the *i*th gene across in *j*th sample; ${\mu }_{j}$ is the mean of the ${\text{log}}_{2}\text{CPM}$ values for *j*th sample across all genes; ${\sigma }_{j}$ is the standard deviation of the ${\text{log}}_{2}\text{CPM}$ values for *j*th sample across all genes.
- Scaled mean: z-score standardization of the mean of expression matrix in terms of log2 CPM
  $${z}_{i}=\frac{\overline{{x }_{i}}-\mu }{\sigma }$$
  where $\overline{{x }_{i}}$ is the mean of the ${\text{log}}_{2}\text{CPM}$ value for the *i*th gene across all samples; $\mu$ is the mean of the overall average ${\text{log}}_{2}\text{CPM}$ values for samples across all genes; $\sigma$ is the standard deviation of mean across all genes.
- Fraction zero: fraction zero per spot.
- Library size vs fraction zero: the relationship between library size and the proportion of zero per gene.
- Sample Pearson correlation
  $${r}_{AB}=\frac{{\sum }_{i=1}^{n}\left({A}_{i}-\overline{A }\right)\left({B}_{i}-\overline{B }\right)}{\sqrt{{\sum }_{i=1}^{n}{\left({A}_{i}-\overline{A }\right)}^{2}}\sqrt{{\sum }_{i=1}^{n}{\left({B}_{i}-\overline{B }\right)}^{2}}}$$
  where ${A}_{i}$ and ${B}_{i}$ are the ${\text{log}}_{2}\text{CPM}$ values of gene $A$ and $B$ in the *i*th sample; $\overline{A }$ and $\overline{B }$ are the mean ${\text{log}}_{2}\text{CPM}$ values of gene $A$ and $B$ across all samples; $n$ is the number of samples.

### Gene-level

- Fraction zero gene: proportion of zero per gene.
- Scaled variance: *z*-score standardization of the variance of expression matrix in terms of log2 CPM
  $${z}_{ij}=\frac{{x}_{ij}-{\mu }_{j}}{{\sigma }_{j}}$$
  where ${x}_{ij}$ is the ${\text{log}}_{2}\text{CPM}$ value for the *i*th sample in the *j*th gene; ${\mu }_{j}$ is the mean of the ${\text{log}}_{2}\text{CPM}$ values for the *j*th gene across all samples; ${\sigma }_{j}$ is the standard deviation of the ${\text{log}}_{2}\text{CPM}$ values for the *j*th gene across all samples.
- Scaled mean: *z*-score standardization of the mean of expression matrix in terms of log2 CPM
  $${z}_{i}=\frac{\overline{{x }_{i}}-\mu }{\sigma }$$
  where $\overline{{x }_{i}}$ is the mean of the ${\text{log}}_{2}\text{CPM}$ value for the *i*th samples across all genes; $\mu$ is the mean of the overall average ${\text{log}}_{2}\text{CPM}$ values for genes across all samples; $\sigma$ is the standard deviation of mean across all samples.
- Mean vs variance: the relationship between mean expression and variance expression.
- Mean vs fraction zero: the relationship between mean expression and the proportion of zero per gene.
- Gene Pearson correlation
  $${r}_{AB}=\frac{{\sum }_{i=1}^{n}\left({A}_{i}-\overline{A }\right)\left({B}_{i}-\overline{B }\right)}{\sqrt{{\sum }_{i=1}^{n}{\left({A}_{i}-\overline{A }\right)}^{2}}\sqrt{{\sum }_{i=1}^{n}{\left({B}_{i}-\overline{B }\right)}^{2}}}$$
  where ${A}_{i}$ and ${B}_{i}$ are the ${\text{log}}_{2}\text{CPM}$ values of sample $A$ and $B$ in the *i*th gene; $\overline{A }$ and $\overline{B }$ are the mean ${\text{log}}_{2}\text{CPM}$ values of sample $A$ and $B$ across all genes; $n$ is the number of genes.

### Spatial-level

This section of our study focuses on spatial-level metrics. We used transition matrix, neighborhood enrichment matrix, and centralized score matrix to depict the global spatial patterns evident in spatial transcriptomic data, originating from spider [9]. The other four metrics (cell type interaction, Moran’s *I*, *L* statistic, and nearest neighbor correlation), originating from scFeatures [16], are specifically designed for a multi-view representation of spatial data and includes feature types commonly used in spatial analysis.

- Transition matrix (TM): this is a normalized matrix representing transition frequencies, which embodies the probabilities of transitioning from one state to another within a Markov chain framework. In this context, the TM elucidates the interrelationships among spatial clusters in each space. Each element in the matrix signifies the transition probability from one spatial cluster to another, thereby mapping the dynamic interplay of spatial clusters.
- Neighborhood enrichment matrix (NEM): This matrix quantifies the enrichment observed between each pair of spatial clusters. It serves to systematically assess the interaction between different clusters within a spatial context, providing insights into the relative connectivity between various spatial clusters
  $${\text{NEM}}_{ij}=\frac{{x}_{ij}-{\mu }_{ij}}{{\sigma }_{ij}},\hspace{1em}i,j=1,\dots ,K$$
  where ${x}_{ij}$ is the number of connections between the group of *i*th spatial cluster ${C}_{i}$ and the group of *j*th spatial cluster ${C}_{j}$; ${\mu }_{ij}$ is the expected mean; ${\sigma }_{ij}$ is the standard deviation.
- Centralized score matrix (CSM)
  $$\text{CSM}=\left[{G}_{1},{G}_{2},{G}_{3}\right]\in {R}^{k\times 3}$$
  where ${G}_{1}$ is group degree centrality; ${G}_{2}$ is the average clustering coefficient; ${G}_{3}$ is the group closeness centrality. Each of the details is shown below:
  - ◦ Group degree centrality: calculates the ratio of spots within one spatial cluster that are connected to spots in another spatial cluster. It assesses the inter-cluster connectivity, indicating the extent to which one cluster is interlinked with another
    $${G}_{1}\left(k\right)=\frac{\left|N\left({C}_{k}\right)\right|}{N-\left|{C}_{k}\right|},\hspace{1em}k=1,\dots ,K$$
    where ${C}_{k}$ is the group of *k*th spatial cluster; $N\left({C}_{k}\right)$ is the neighbors of all the spot in ${C}_{k}$; $N$ is the number of spots.
  - ◦ Average clustering coefficient: measures the propensity for a spot within a spatial cluster to be connected to spots in another cluster. It provides insights into the likelihood of inter-cluster associations, reflecting the tendency of spots to form connections beyond their immediate cluster
    $${G}_{2}\left(k\right)=\frac{1}{\left|{C}_{k}\right|}{\sum }_{v\in {C}_{k}}\frac{2{t}_{v}}{{d}_{v}\left({d}_{v}-1\right)},\hspace{1em}k=1,\dots ,K$$
    where ${t}_{v}$ is the number of triangles around spot $v$; ${C}_{k}$ is the group of *k*th spatial cluster; ${d}_{v}$ is the degree of spot $v$, which is the number of connections or edges.
  - ◦ Group closeness centrality: the normalized inverse sum of distances from a spatial cluster to all spots in a different spatial cluster. It quantifies the relative proximity or accessibility of one cluster to all spots in another, offering a measure of how closely or centrally positioned a cluster is concerning another cluster in the spatial arrangement
    $${G}_{3}\left(k\right)=\frac{\left|V-{C}_{k}\right|}{\sum_{v\in V-{C}_{k}}dist\left(v,{C}_{k}\right)},\hspace{1em}k=1,\dots ,K$$
    where *V* is the number of spot; ${C}_{k}$ is the group of *k*th spatial cluster; $dist\left(v,{C}_{k}\right)$ is the shortest distance between spatial cluster ${C}_{k}$ and spot $v$.
- Cell type interaction: assume the nearest neighbors should be the cells captured within each spot and consider them as the spatial interaction pairs. Then used the estimated cell type proportion in each spot to calculate the spatial interaction between cell types. For details refer to scFeatures.
- Moran’s *I*: measure spatial autocorrelation, meaning how strongly the feature expression value in a sample cluster or disperse
  $$\text{Moran's}\,I=\frac{N}{W}\frac{\sum_{i=1}^{N}\sum_{j=1}^{N}{w}_{ij}\left({x}_{i}-\overline{x }\right)\left({x}_{j}-\overline{x }\right)}{\sum_{i=1}^{N}{\left({x}_{i}-\overline{x }\right)}^{2}}$$
  $$\text{where }{w}_{ij}\left\{\begin{array}{c}1, if i and j are spatial neighbors \\ 0, else\end{array}\right.,\hspace{1em}W={\sum }_{i,j}{w}_{ij}$$
  where ${x}_{i}$ and ${x}_{j}$ are the gene expression values of *i*th cell and *j*th cell; $\overline{x }$ is the average gene expression value of one gene; $N$ is the number of cells.

- *L* statistics: the *L* value between the pairs of genes by estimation cell type proportion. For details refer to scFeatures.
- Nearest neighbor correlation: Pearson correlation for gene expression between a spot with its nearest neighbor spot. For details refer to scFeatures.

#### Evaluation of spatial downstream analysis

This evaluation is spatial downstream analysis including spatial clustering, cell type deconvolution, spatially variable gene identification, and spatial cross-correlation. We performed each algorithm on the real experimental dataset and simulated dataset and compared the similarity of the result.

### Spatial clustering

Spatial clustering refers to cluster or group spots based on similar expression patterns across a spatial domain. We apply adjusted Rand index (ARI) and normalized mutual information (NMI) to evaluate the spatial clustering result between real data and simulated data.

- Adjusted Rand index (ARI): measure the similarity between two clusters in real and simulated datasets
  $$\text{ARI}=\frac{{\sum }_{ij}\left(\genfrac{}{}{0pt}{}{{n}_{ij}}{2}\right)-\left[{\sum }_{i}\left(\genfrac{}{}{0pt}{}{{a}_{i}}{2}\right){\sum }_{j}\left(\genfrac{}{}{0pt}{}{{b}_{j}}{2}\right)\right]/\left(\genfrac{}{}{0pt}{}{n}{2}\right)}{\frac{1}{2}\left[{\sum }_{i}\left(\genfrac{}{}{0pt}{}{{a}_{i}}{2}\right)+{\sum }_{j}\left(\genfrac{}{}{0pt}{}{{b}_{j}}{2}\right)\right]-\left[{\sum }_{i}\left(\genfrac{}{}{0pt}{}{{a}_{i}}{2}\right){\sum }_{j}\left(\genfrac{}{}{0pt}{}{{b}_{j}}{2}\right)\right]/\left(\genfrac{}{}{0pt}{}{n}{2}\right)}$$
  where *n* is the total number of spots; ${a}_{i}$ is the number of items in the *i*th spatial cluster of the spatial clustering in real dataset; ${b}_{j}$ is the number of items in the *j*th spatial cluster of the spatial clustering in simulated dataset; ${n}_{ij}$ is the number of items that are in both the *i*th cluster of the spatial clustering in real dataset and the *j*th cluster of the spatial clustering in a simulated dataset.
- Normalized mutual information (NMI): a measure of the mutual dependence between the real and simulated spatial clusters
  $$\text{NMI}=\frac{{\sum }_{i,j}{p}_{ij}\text{log}\left(\frac{{p}_{ij}}{{p}_{i}{p}_{j}}\right)}{\frac{1}{2}\left(-{\sum }_{i}{p}_{i}\text{log}{p}_{i}-{\sum }_{j}{p}_{j}\text{log}{p}_{j}\right)}$$
  $$\text{where}\hspace{1em}{p}_{ij}=\frac{{n}_{ij}}{n},\hspace{1em}{p}_{i}=\frac{{n}_{i}}{n},\hspace{1em}\text{and}\hspace{1em}{p}_{j}=\frac{{n}_{j}}{n}$$
  where $n$ is the total number of spots; ${n}_{i}$ is the number of items in the *i*th spatial cluster of the spatial clustering in real dataset; ${n}_{j}$ is the number of items in the *j*th spatial cluster of the spatial clustering in simulated dataset; ${n}_{ij}$ is the number of items that are in both the *i*th cluster of the spatial clustering in real dataset and the *j*th cluster of the spatial clustering in a simulated dataset.

### Cell type deconvolution

Cell type deconvolution refers to interpreting mixed signals within tissue compartments to identify proportions of cell types per spot. This is only relevant to spot-based technology where you have multiple cells per spot. However, this is not relevant for a single-cell based platform where you are able to measure the expression for a single cell. Here, we use the CARD package to perform cell type deconvolution.

We assume that the number of genes per spot is $J$ and the number of spot is $I$ in spatial transcriptomics data; ${X}_{ij}$ represent the spatial gene expression value of gene $j$ in the *i*th spot; ${T}_{ik}$ and ${P}_{ik}$ are the true and predicted proportion of cell type $k$.

- RMSE: root mean square error is calculated between ${T}_{ik}$ and ${P}_{ik}$ of per cell type. After that, we normalize them by sum of proportions among all the spots ${S}_{k}$. Then, we average all the RMSE as final RMSE

$${\text{RMSE}}=\sqrt{\frac{1}{K}{\sum }_{k=1}^{K}\frac{1}{{S}_{k}}{\sum }_{i=1}^{I}{\left({P}_{ik}-{T}_{ik}\right)}^{2}}$$

- JSD: We use Kullback–Leibler divergence (KL) to calculate JSD. $Q\left({T}_{k}\right)$ and $Q\left({P}_{k}\right)$ are true distribution and algorithm-predicted distribution of cell type $k$. Then, we average all the JSD as final JSD

$$\text{JSD}=\frac{1}{2}\text{KL}\left(Q\left({T}_{k}\right) || \frac{Q\left({P}_{k}\right)+Q\left({T}_{k}\right)}{2}\right)+\frac{1}{2}\text{KL}\left(Q\left({P}_{k}\right) ||\frac{Q\left({P}_{k}\right)+Q\left({T}_{k}\right)}{2}\right)$$

$$\text{KL}\left(Q\left({P}_{k}\right) || Q\left({T}_{k}\right)\right)=\sum Q\left({P}_{k}\right)\text{In}\frac{Q\left({P}_{k}\right)}{Q\left({T}_{k}\right)}$$

### Spatially variable gene (SVG) identification

Spatially variable gene identification refers to identifying genes whose expression levels vary significantly across different spatial coordinates or regions. We apply precision and recall to evaluate the results.

- Precision: the proportion of correctly identified items in simulated datasets
  $${\text{Precision}}=\frac{\text{TP}}{\text{TP}+\text{FP}}$$
  where false positive (FP) is the number of genes that are incorrectly identified as SVG when they are not SVG; true positive (TP) is the number of genes that are correctly identified as SVG.
- Recall: the proportion of real SVG correctly identified in the simulated dataset
  $${\text{Recall}}=\frac{\text{TP}}{\text{TP}+\text{FN}}$$
  where false negative (FN) is the number of genes that are incorrectly predicted as non-SVG when they are true SVG.

### Spatial cross-correlation

Spatial cross-correlation explores how two distinct genes co-vary spatially by bivariate Moran’s *I*. We apply cosine similarity and Mantel statistics to evaluate the results.

- Bivariate Moran’s *I*: Moran’s *I* between genes $X$ and $Y$ was calculated by the following:
  $${I}_{XY}=\frac{N}{W}\frac{{\sum }_{i}{\sum }_{j}{w}_{ij}\left({x}_{i}-\overline{x }\right)\left({y}_{j}-\overline{y }\right)}{\sqrt{{\sum }_{i}{\left({x}_{i}-\overline{x }\right)}^{2}}\sqrt{{\sum }_{i}{\left({y}_{i}-\overline{y }\right)}^{2}}}$$
  $$\text{where }{w}_{ii}=0,\hspace{1em}W=\sum {w}_{ij}$$
  where ${w}_{ij}$ represents a spatial weight matrix; $N$ represents the number of spots; ${x}_{i}$ and ${y}_{i}$ are the *i*th components of gene $X$ and $Y$; $\overline{x }$ is the mean of all elements ${x}_{i}$; $\overline{y }$ is the mean of all elements ${y}_{i}$.
- Cosine similarity: measure similarity between bivariate Moran’s *I* of real dataset $A$ and that of in simulation dataset $B$
  $$\text{cosine similarity}={S}_{C}\left(A,B\right):=\text{cos}\left(\theta \right)=\frac{A\cdot B}{||A||||B||}=\frac{{\sum }_{i=1}^{n}{A}_{i}{B}_{i}}{\sqrt{{\sum }_{i=1}^{n}{A}_{i}^{2}}\cdot \sqrt{{\sum }_{i=1}^{n}{B}_{i}^{2}}}$$
  where ${A}_{i}$ and ${B}_{i}$ are the *i*th components of vectors $A$ and $B$, respectively.
- Mantel statistics: The test statistic for the Mantel test, which is a correlation coefficient calculated between bivariate Moran’s *I* of real dataset $A$ and that of in simulation dataset $B$
  $$r=\frac{{\sum }_{i=1}^{n}{\sum }_{j=1}^{n}\left({A}_{ij}-\overline{A }\right)\left({B}_{ij}-\overline{B }\right)}{\sqrt{{\sum }_{i=1}^{n}{\sum }_{j=1}^{n}{\left({A}_{ij}-\overline{A }\right)}^{2}{\sum }_{i=1}^{n}{\sum }_{j=1}^{n}{\left({B}_{ij}-\overline{B }\right)}^{2}}]}$$
  where ${A}_{ij}$ is the element in the *i*th spot and *j*th gene of $A$; ${B}_{ij}$ is the element in the *i*th spot and *j*th gene of $B$; $\overline{A }$ is the mean of all elements ${A}_{ij}$ in dataset $A$; $\overline{B }$ is the mean of all elements ${B}_{ij}$ in dataset $B$; $n$ is the number of spots in $A$ or $B$.

### Ranking consistency by concordance index

For spatial clustering and spatially variable gene (SVG) identification tasks, we applied multiple methods to analyze both real and simulated datasets. Specifically, we utilized five SVG identification methods (SPARK-X [18], nnSVG [19], MERINGUE [20], Seurat’s HVG [21], and Giotto [22]) and six spatial clustering methods (BayesSpace, Seurat’s Leiden [21], PRECAST [23], DR.SC [24], BASS [25], and SpatialPCA [26]). Detailed descriptions of these tools are provided in Additional file 1: Table S2 and Additional file 1: Table S3. The concordance index, which quantifies alignment between real and simulated data outcomes, was computed to compare the evaluation results. Consistency was analyzed in two layers: the first layer examined concordance across tools within each spatial task and different simulation models, leading to a ranking of simulation methods. The second layer then assessed the correlation between these rankings and the method rankings established in previous results, as shown in Fig. 4.

In SVG identification tasks, we evaluated results by calculating the proportion of SVGs as a key metric. For all methods except Seurat’s HVG, we used a threshold of an adjusted *P* value below 0.05; for Seurat’s HVG, the threshold was set at the median of standardized variance and mean. In the spatial clustering tasks, we used the ARI and NMI metrics for evaluation.

- Concordance index: the C-index measures the agreement results between true and simulated outcome, where a higher C-index indicates better alignment between the simulated ranking and the true ranking of the datasets
  $$\text{C-index}=\frac{1}{\left|\{\left(i,j\right):{T}_{i}\ne {T}_{j}\}\right|}{\sum }_{i<j}\delta \left({T}_{i}\ne {T}_{j}\right)\delta \left(\widehat{{T}_{i}}<\widehat{{T}_{j}}\right)$$
  where ${T}_{i}$ and ${T}_{j}$ are true outcomes and $\widehat{{T}_{i}}$ and $\widehat{{T}_{j}}$ are the corresponding predicted values. The C-index sums over all pairs $\left(i,j\right)$ where the true outcomes differ, counting cases where the predicted order aligns with the true order. The result is normalized by the total number of comparable pairs.

#### Evaluation of method comparison in each score and rank-based overall score

In order to summarize the results derived from multiple datasets and criteria, we employed a multi-step approach to generate scores. This process was essential due to the utilization of distinct metrics, with KDE test statistics being applied for both spot-wise and gene-wise evaluations, as well as a part of the spatial pattern assessments. Also, spatial clustering and some spatial pattern evaluation utilized different scoring systems, necessitating a structured method to integrate these diverse scores coherently. Details are shown below.

In a kernel density estimation (KDE) test, the null hypothesis assumes that the two estimated densities are identical. The integrated squared error (ISE) quantifies the discrepancy between these estimates. Under the null hypothesis, the final test statistic is calculated based on the ISE:

$$T=\int {\left[{f}_{1}\left(x\right)-{f}_{2}\left(x\right)\right]}^{2}dx$$

where ${f}_{1}\left(x\right)$ and ${f}_{2}\left(x\right)$ are the kernel density estimates of sample 1 and sample 2, respectively. The $T$ statistics is then adjusted for variance and bias, leading to a standardized test statistic ${\text{KD}}{\text{E}}_{z}$:

$${\text{KDE}}_{z}=\frac{T-{\mu }_{T}}{{\sigma }_{T}}$$

where $${\mu }_{T}$$ is the mean of $$T$$ under the null hypothesis; $${\sigma }_{T}$$ is the standard deviation of $$T$$ under the null hypothesis. This is implemented in the function kde.test() inside the R package ks (v1.14.2). Here, a small value of $$T$$ and $${\text{KDE}}_{z}$$ both indicate better performance; we extracted *z*-test statistics using the following command: kde.test()$zstat.

To further adjust the difference in distribution between the variables (e.g., fraction zero, library size), we transformed this *z* statistic into a similarity measure ranging from 0 (perfectly similar) to 1 (completely dissimilar) using the below equation:

$${\text{KDE}}_{z{\text{transformed}}}=\frac{{\text{KDE}}_{z}-{\text{KDE}}_{z\text{min}}}{{\text{KDE}}_{z\text{max}}-{\text{KDE}}_{z\text{min}}}$$

where ${\text{KDE}}_{z}$ is the raw value before the transformation, ${\text{KDE}}_{z\text{min}}$ is the minimum value, and ${\text{KDE}}_{z\text{max}}$ is the maximum value before transformation. This transformation is applied to the values obtained from all methods across all datasets for each variable. To ensure consistency, ${\text{KDE}}_{z\text{min}}$ and ${\text{KDE}}_{z\text{max}}$ are defined based on the range of all these ${\text{KDE}}_{z}$ in each variable.

Our initial step involved generating individual KDE test statistics for each dataset via each simulation model. This allowed us to summarize the performance of each method across all datasets by calculating their average scores. This resulted in a single score of comparison among all methods.

Following this, we established an overarching score for each method by integrating the metrics to each method’s evaluation across datasets. For instance, in calculating a rank-based overall score focused on spot-wise evaluation, we first computed the KDE test statistics for each method across nine metrics. Then, we arranged the spot-wise metrics of these methods in ascending order—effectively ranking them from best to worst—across nine metrics. The final step involved averaging these rankings to obtain an overall accuracy score, as follows:

$$\text{Overall score (spot-wise)}=\frac{1}{9}\left({\text{rank}}_{\text{fracZero}}+{\text{rank}}_{\text{libSize}}+{\text{rank}}_{\text{TMM}}+\dots +{\text{rank}}_{\text{scaledMean}}\right)$$

### Evaluation of impact of dataset distribution in method performance

To synthesize the outcomes from various criteria across four assessments, we adopted a layered method to calculate scores for every dataset. Initially, we divided the process into four distinct evaluations: spot-wise, gene-wise, and spatial patterns, recognizing that each assessment captures unique aspects.

In the subsequent phase, we computed the average of scores from several metrics for each dataset within every evaluation. We then determined the overall score for each dataset across the simulation model in all four evaluations. For instance, in assessing the consistency of a dataset within the spot-wise evaluation, we averaged the KDE test statistics from nine different criteria for a single dataset using one method.

### Evaluation of impact of model distribution in method performance

To assess the impact of models within a specific metric, we performed linear regression analyses for each metric separately. This approach allowed us to quantify the effect of switching from one model to another within the same metric context. By comparing the regression coefficients, we could determine which models had statistically significant impacts on the scores, thereby evaluating each model’s effectiveness across different metrics. This analysis was conducted using the lm function in the built-in stats (v4.4.1) package in R.

For each metric, a linear regression model was fitted where the dependent variable was a KDE test statistics, and the independent variable was the type of model with the formula defined as:

$${y}_{i}={\beta }_{0}+{\beta }_{1}{X}_{1,i}+{\beta }_{2}{X}_{2,i}+\dots +{\beta }_{k}{X}_{k,i}+{\epsilon }_{i}$$

where ${y}_{i}$ represents score for the *i*th observation; ${X}_{1,i},{X}_{2,i},\dots ,{X}_{k,i}$ are indicator variables (dummy variables) derived from the model factor, representing the presence (1) or absence (0) of each category (excluding the reference category) for the *i*th observation; ${\beta }_{0}$ is the intercept term; and ${\beta }_{1},{\beta }_{2},\dots ,{\beta }_{k}$ are the regression coefficients for each of the indicator variables.

### Evaluation of scalability

To mitigate potential confounding effects, our analysis of scalability was confined to Dataset 7, which we systematically downsampled to produce datasets with varying numbers of spots and genes, specifically including spot counts of 200, 500, 1000, 3000, and 5000, and gene counts of 200, 500, and 1000, resulting in 15 downsampled datasets.

The execution time for each method was gauged using the Sys.time function in R and the time.time function in Python. Tasks failing to complete within the allotted time frame were deemed to have generated no results. To capture the peak memory usage of R methods, we utilized the psutil library to monitor the maximal resident set size, with all measurements taken thrice and averaged for accuracy.

In the simAdaptor approach, we implemented a two-step simulation process: initially conducting spatial clustering on the dataset and labeling each spot, followed by sequentially integrating each spatial cluster into the simulation models. The duration and memory consumption for both stages were recorded separately and are presented in Additional file 1: Fig. S4. Conversely, for one-step approaches where the entire dataset is fed into the simulation model simultaneously, we only tracked the time and memory requirements of this singular process.

Computational resources for these tests included running the 13 simulation methods on an R server equipped with Intel Core i9-14900K CPUs (5.2 GHz, 36 MB Smart Cache, and a total of 24 CPU cores) and 64 GB of DDR5 6000 MHz memory.

## Supplementary Information

Supplementary Material 1

## Data Availability

All datasets used in this study are publicly available. Details on each datasets, including their accession ID are provided in Additional File 1: Table S2. Curated version of the datasets is available in Figshare (10.6084/m9.figshare.26054188.v3) [38] and is deposited in Zenodo (https://zenodo.org/records/14777824) [39]. Below we provide the accession numbers when available or download links used to obtain each dataset. • Dataset 1: Wu et al., [41]. Human breast cancer (CID3586). Downloaded from GEO accession GSE176078 [42]. • Dataset 2: Spatial data: Xia et al., [43]. Human osteosarcoma. Downloaded from the supplementary section of the corresponding paper. https://www.pnas.org/doi/suppl/10.1073/pnas.1912459116/suppl_file/pnas.1912459116.sd12.csv. Single cell data: Zhou et al., [44]. Human osteosarcoma (BC22). Downloaded from GEO accession GSE152048 [45]. • Dataset 3: McCray et al., [46]. Human prostate. Downloaded from GEO accession GSE159697 [47]. • Dataset 4: Kleshchevnikov et al., [48]. Mouse brain. Downloaded from https://github.com/BayraktarLab/cell2location • Dataset 5: Lopez et al., [49]. Mouse fibrosarcoma. Downloaded from https://github.com/romain-lopez/DestVI-reproducibility • Dataset 6: Eng et al., [25]. Mouse cortex. Downloaded from https://github.com/CaiGroup/seqFISH-PLUS • Dataset 7: Lohoff T et al. [50], Mouse gastrulation. Downloaded from https://content.cruk.cam.ac.uk/jmlab/SpatialMouseAtlas2020/ • Dataset 8: Ståhl et al., Mouse olfactory bulb. Downloaded from the supplementary section of the corresponding paper. www.spatialtranscriptomicsresearch.org • Dataset 9: McKellar et al., Mouse hindlimb muscle. Downloaded from GEO accession GSE161318 [51]. • Dataset 10: Moncada et al., Human pancreatic ductal adenocarcinomas. Downloaded from GEO accession GSE111672 [52].

## Code availability

SpatialSimBench were run on R (v4.3) or python (v3.8) and the source code is deposited in Zenodo (https://zenodo.org/records/14777824) [39] and is freely available from Github (https://github.com/SydneyBioX/SpatialSimBench) [40]. The SpatialSimBench website provides an interactive platform for users to sort and explore results based on various performance metrics. This web tool, developed using Quarto (v1.5.56) with Observable JavaScript (OJS) and R, is available at https://sydneybiox.github.io/SpatialSimbench_website/.
