# Supplementary material for: Multi-task benchmarking of spatially resolved gene expression simulation models
Source: Genome Biol. 2025 Mar 17;26:57. doi: 10.1186/s13059-025-03505-w (PMC11912772; doi:10.1186/s13059-025-03505-w)
Supplement: Supplementary file 1 — Supplementary Material 1 [file 13059_2025_3505_MOESM1_ESM.docx]

Multi-task benchmarking of spatially resolved gene expression simulation models

Xiaoqi Liang^1,2,3^, Marni Torkel^1,2,3^,Yue Cao ^1,2,3,4+^, Jean Yee Hwa Yang^1,2,3,4^*^+^

^1^ School of Mathematics and Statistics, The University of Sydney, NSW 2006, Australia.
^2^ Sydney Precision Data Science Centre, The University of Sydney, NSW 2006, Australia

^3^ Charles Perkins Centre, The University of Sydney, NSW 2006, Australia.

^4^ Laboratory of Data Discovery for Health Limited (D^2^4H), Science Park, Hong Kong SAR, China.

^+^Contributed equally

*Co-Correspondence to [jean.yang@sydney.edu.au](mailto:jean.yang@sydney.edu.au) and [yue.cao@sydney.edu.au](mailto:yue.cao@sydney.edu.au)

**Supplementary figures: Figs S1-S5.**

###
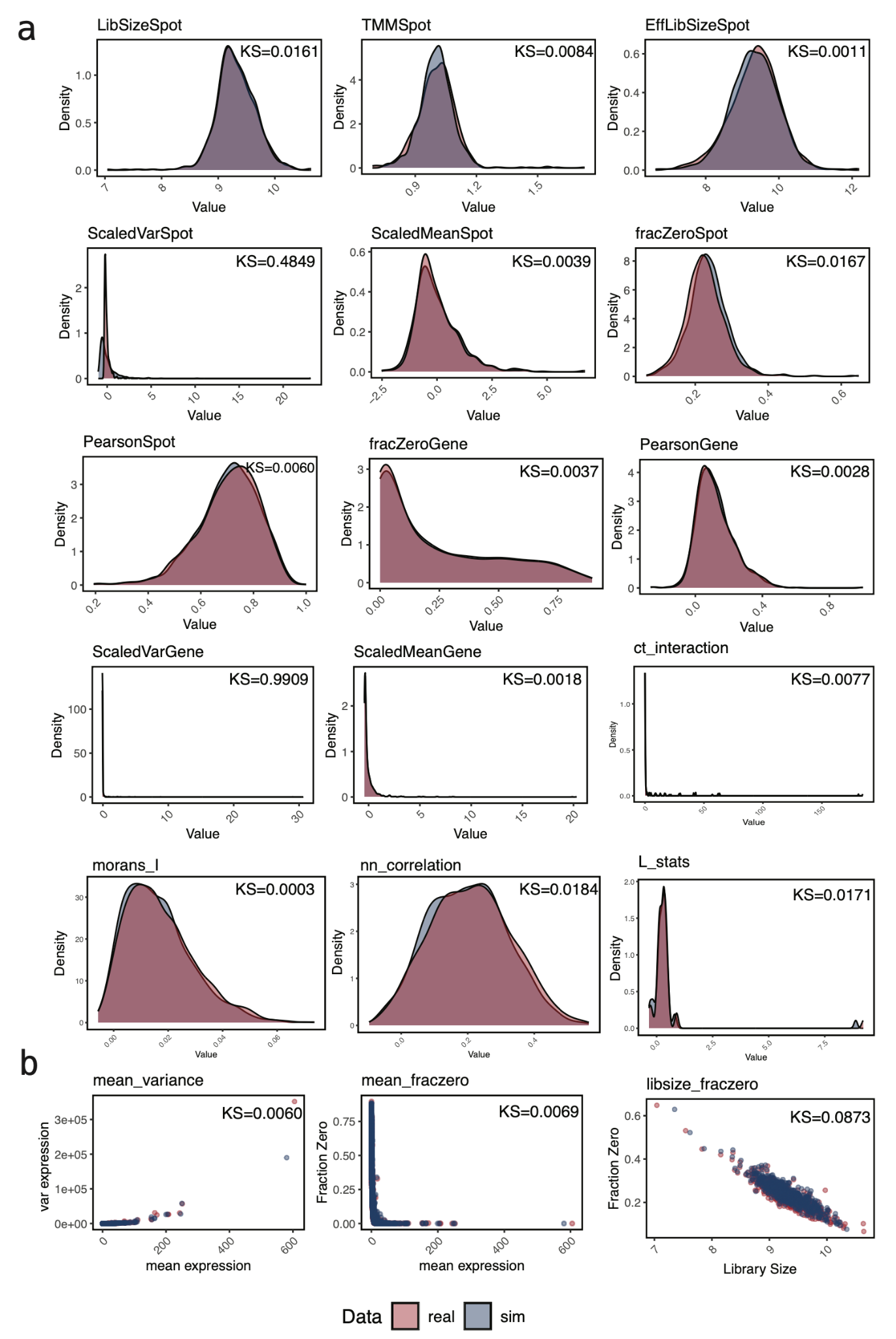


**Figure S1. SRTsim evaluation of each data property across Spot-level, Gene-level and Spatial-level in Dataset 2. a** One dimension visualized by density plot. **b** Bivariate visualized by scatter plot. KS is the KDE test statistics.

**
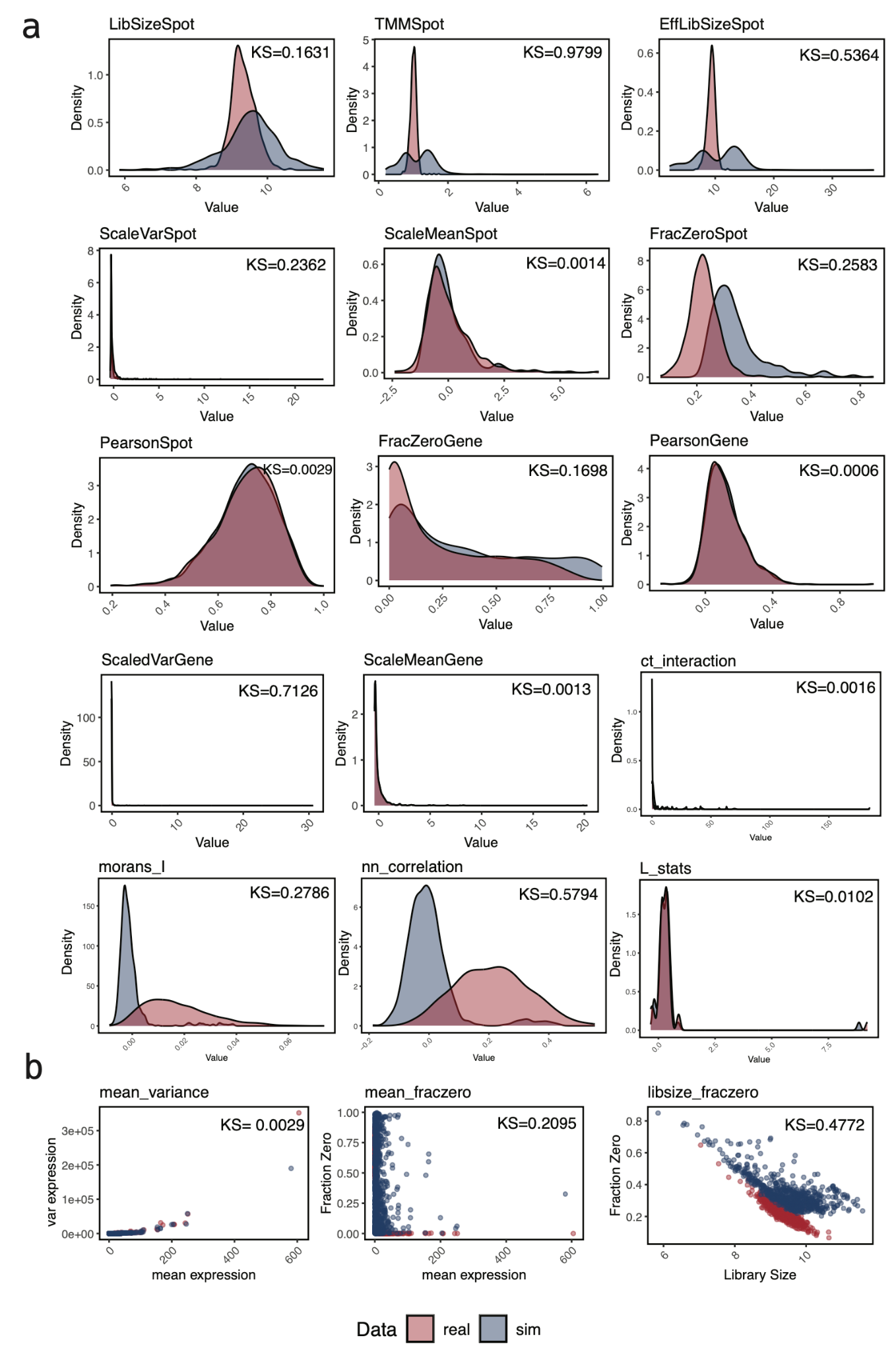
**

**Figure S2. Symsim evaluation of each data property across Spot-level, Gene-level and Spatial-level in Dataset 2. a** One dimension visualized by density plot. **b** Bivariate visualized by scatter plot. KS is the KDE test statistics.


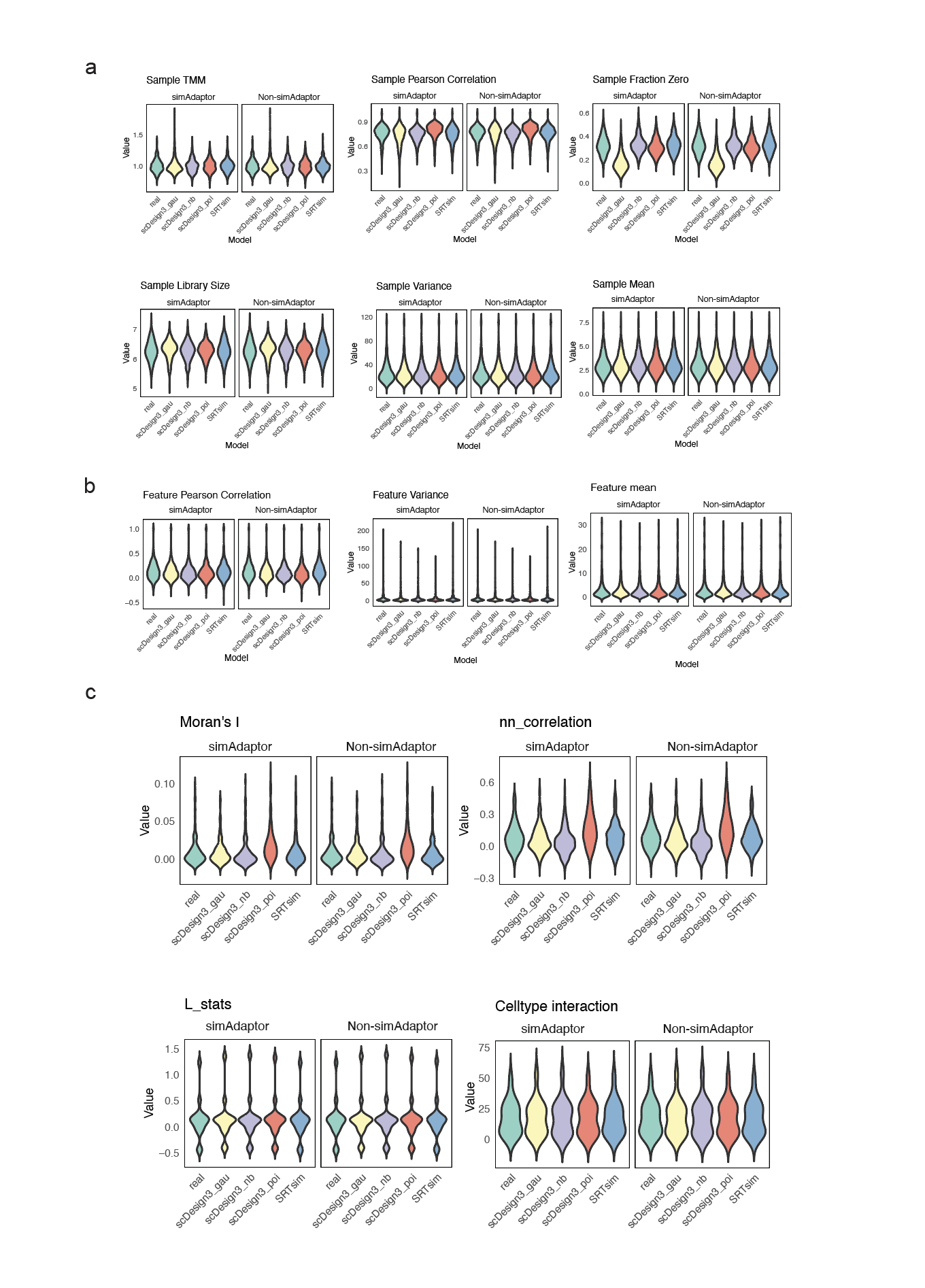


**Figure S3. Comparison of approaches based on using simAdaptor and those without simAdaptor, with an assessment of data properties results from spatial simulators.** **a** Visualizations of the real and simulation in boxplot across spot-level metrics. **b** Visualizations of the real and simulation in boxplot across gene-level metrics. **c** Visualizations of the real and simulation in boxplot across spatial-level metrics.

####


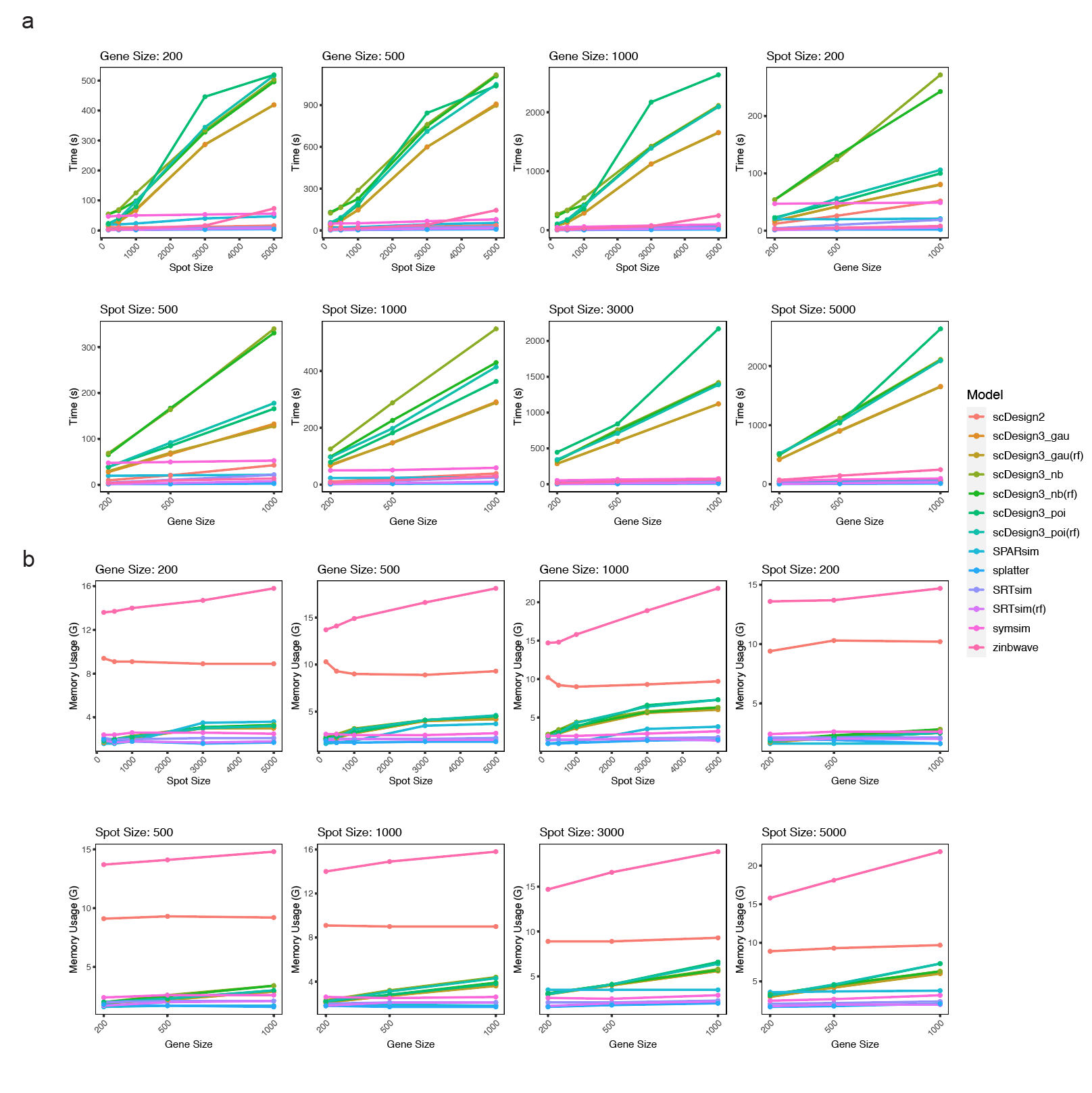


**Figure S4. Run time and memory consumption of each method across various spot sizes and gene sizes.** **a** Runtime of each method across spot size and gene size. **b** Maximal memory usage of each method across spot size and gene size.

**
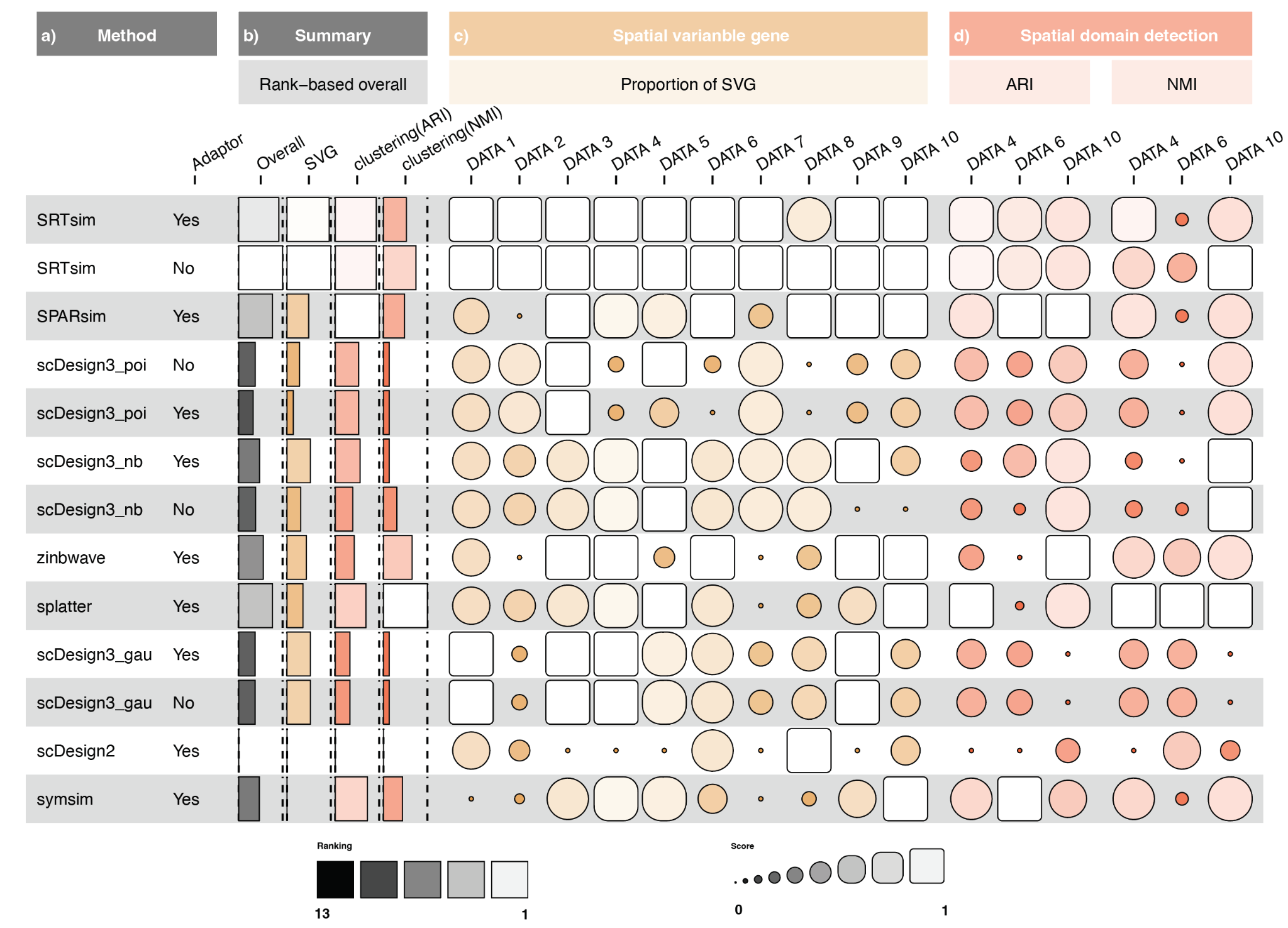
**

**Figure S5. Concordance of ranking consistency.** The colour represents different areas of evaluation and the higher score shows the best possible rank of 1. **a** The name of the method across Non-simAdaptor and simAdaptor approaches. **b** Summary of all the overall performance. **c** Score of method within evaluation of spatial variable gene (SVG) identification, ranking by the proportion of SVG. **d** Score of methods within spatial domain detection, ranking by ARI and NMI.

**Supplementary tables: Table S1-S5.**

**Table S1.** Details of the spatial and single-cell simulation methods evaluated in this study.

| Methods | Year of publication | Package version | Parameter setting | Spatial information required |
| --- | --- | --- | --- | --- |
| scDesign3_nb | 2023 | 1.1.1 | family_use = 'nb', others are default | Yes |
| scDesign3_poi | 2023 | 1.1.1 | family_use = 'poi', others are default | Yes |
| scDesign3_gau | 2023 | 1.1.1 | family_use = 'gaussian', others are default | Yes |
| SRTsim | 2023 | 0.99.6 | default | Yes |
| Splatter | 2017 | 1.26.0 | default | No |
| ZINB-WaVE | 2018 | 1.24.0 | default | No |
| SymSim | 2019 | 0.0.0.9000 | default | No |
| scDesign2 | 2021 | 0.1.0 | default | No |
| SPARsim | 2020 | 0.9.5 | default | No |

**Table S2.** Details of the spatially variable genes identification methods evaluated in this study.

| Methods | Year of publication | Package version | Parameter setting | Ranking statistics |
| --- | --- | --- | --- | --- |
| SPARK-X | 2021 | 1.1.1 | default | Adjusted p-value |
| nnSVG | 2023 | 1.8.0 | default | Log-Likelihood ratio statistic |
| MERINGUE | 2021 | 1.0.0 | default | Observed coefficient |
| HVG by Seurat | 2018 | 5.1.0 | default | Standardized variance |
| Giotto | 2021 | 4.1.0 | default | Adjusted p-value |

**Table S3.** Details of the spatial clustering methods evaluated in this study.

| Methods | Year of publication | Package version | Parameter setting |
| --- | --- | --- | --- |
| BayesSpace | 2021 | 1.14.0 | The number of spatial domains sets to the true number; others are recommended settings. |
| Leiden by Seurat | 2018 | 5.1.0 |  |
| PRECAST | 2023 | 1.6.5 |  |
| DR.SC | 2022 | 3.4 |  |
| BASS | 2022 | 1.3.1 |  |
| SpatialPCA | 2022 | 1.3.0 |  |

**Table S4.** Details of the spatial gene expression data and single-cell data evaluated in this study.

| Dataset | Data modality | Species | Tissue | Health state | Protocol | Spot number | Gene number | Source | Data source paper |
| --- | --- | --- | --- | --- | --- | --- | --- | --- | --- |
| Dataset 1 | Spatial | Human | breast | cancer | Visium | 4744 | 28402 | CID3586 [GSE176078](https://www.ncbi.nlm.nih.gov/geo/query/acc.cgi?acc=GSE176078) | A single-cell and spatially resolved atlas of human breast cancers [[38]](https://paperpile.com/c/nG5Qy8/DElv) |
|  | Single cell | Human | breast | cancer | Chromium | 6178 | 21164 |  |  |
| Dataset 2 | Spatial | Human | osteosarcoma | normal | MERFISH | 645 | 12903 | [Link](https://www.pnas.org/doi/suppl/10.1073/pnas.1912459116/suppl_file/pnas.1912459116.sd12.csv) | Spatial transcriptome profiling by MERFISH reveals subcellular RNA compartmentalization and cell cycle-dependent gene expression [[39]](https://paperpile.com/c/nG5Qy8/ZDAsa) |
|  | Single cell | Human | osteosarcoma | normal | Chromium | 9234 | 19098 | BC22  [GSE152048](https://www.ncbi.nlm.nih.gov/geo/query/acc.cgi?acc=GSE152048) | Single-cell RNA landscape of intratumoral heterogeneity and immunosuppressive microenvironment in advanced osteosarcoma [[40]](https://paperpile.com/c/nG5Qy8/hbx88) |
| Dataset 3 | Spatial | Human | prostate | normal | Visium | 277 | 36601 | [GSE159697](https://www.ncbi.nlm.nih.gov/geo/query/acc.cgi?acc=GSE159697) | Vitamin D sufficiency enhances differentiation of patient-derived prostate epithelial organoids [[41]](https://paperpile.com/c/nG5Qy8/Zq13t) |
|  | Single cell | Human | prostate | normal | Chromium | 4740 | 27400 |  |  |
| Dataset 4 | Spatial | Mouse | brain | normal | Visium | 2577 | 31053 | [Link](https://github.com/BayraktarLab/cell2location) | Cell2location maps fine-grained cell types in spatial transcriptomics [[42]](https://paperpile.com/c/nG5Qy8/25NGu) |
|  | Single cell | Mouse | brain | normal | Chromium | 40532 | 12820 |  |  |
| Dataset 5 | Spatial | Mouse | fibrosarcoma | tumor | Visium | 2125 | 15976 | [Link](https://github.com/romain-lopez/DestVI-reproducibility) | Multi-resolution deconvolution of spatial transcriptomics data reveals continuous patterns of inflammation [[43]](https://paperpile.com/c/nG5Qy8/zQXVX) |
|  | Single cell | Mouse | fibrosarcoma | tumor | Chromium | 7185 | 25069 |  |  |
| Dataset 6 | Spatial | Mouse | cortex | normal | seqFISH+ | 4744 | 28402 | [Github](https://github.com/CaiGroup/seqFISH-PLUS) | Transcriptome-scale super-resolved imaging in tissues by RNA seqFISH [[27]](https://paperpile.com/c/nG5Qy8/Rw6ec) |
|  | Single cell | Mouse | cortex | normal | Smart-seq | 14249 | 34041 |  |  |
| Dataset 7 | Spatial | Mouse | gastrulation | normal | seqFISH | 8425 | 351 | [Link](https://content.cruk.cam.ac.uk/jmlab/SpatialMouseAtlas2020/) | Integration of spatial and single-cell transcriptomic data elucidates mouse organogenesis [[44]](https://paperpile.com/c/nG5Qy8/VzKmK) |
|  | Single cell | Mouse | gastrulation | normal | Chromium | 4651 | 19103 |  |  |
| Dataset 8 | Spatial | Mouse | olfactory bulb | normal | ST | 278 | 182 | [Link](http://www.spatialtranscriptomicsresearch.org) | Visualization and analysis of gene expression in tissue sections by spatial transcriptomics [[29]](https://paperpile.com/c/nG5Qy8/Y6NuM) |
|  | Single cell | Mouse | olfactory bulb | normal | Chromium | 12640 | 182 |  |  |
| Dataset 9 | Spatial | Mouse | hindlimb muscle | normal | Visium | 884 | 982 | [GSE161318](https://www.ncbi.nlm.nih.gov/geo/query/acc.cgi?acc=GSE161318) | Large-scale integration of single-cell transcriptomic data reveals rare, transient muscle progenitor cell states in muscle regeneration [[45]](https://paperpile.com/c/nG5Qy8/tiL7W) |
|  | Single cell | Mouse | hindlimb muscle | normal | Chromium | 4748 | 14360 |  |  |
| Dataset 10 | Spatial | Human | pancreatic ductal adenocarcinomas | normal | ST | 428 | 25753 | [GSE111672](https://www.ncbi.nlm.nih.gov/geo/query/acc.cgi?acc=GSE111672) | Integrating microarray-based spatial transcriptomics and single-cell RNA-seq reveals tissue architecture in pancreatic ductal adenocarcinomas [[46]](https://paperpile.com/c/nG5Qy8/cgLDZ) |
|  | Single cell | Human | pancreatic ductal adenocarcinomas | normal | Chromium | 1926 | 19736 |  |  |

**Table S3.** Details of the three evaluations categories with metric and range in this study.

| Evaluation | Task | | Transformation | Metric | Range |
| --- | --- | --- | --- | --- | --- |
| Data properties | Spot-level | Fraction zero | No | Kernel Density Scores | [0, 1] |
|  |  | Library size | No |  |  |
|  |  | TMM | No |  |  |
|  |  | Effective library size | No |  |  |
|  |  | Scaled variance | Yes, z-score standardization |  |  |
|  |  | Scaled mean | Yes, z-score standardization |  |  |
|  |  | Library size vs Fraction zero | No |  |  |
|  |  | Sample Pearson correlation | No |  |  |
|  | Gene-level | Fraction zero gene | No |  |  |
|  |  | Scaled variance | Yes, z-score standardization |  |  |
|  |  | Scaled mean | Yes, z-score standardization |  |  |
|  |  | Mean vs variance | No |  |  |
|  |  | Mean vs variance (scale) | Yes, log-normal transformation |  |  |
|  |  | Mean vs fraction zero | No |  |  |
|  |  | Gene Pearson correlation | No |  |  |
|  | Spatial-level | Transition Matrix (TM) | No |  |  |
|  |  | Neighborhood enrichment matrix (NEM) | No |  |  |
|  |  | Centralized score matrix (CSM) | No |  |  |
|  |  | Cell type interaction | No |  |  |
|  |  | Moran’s I | No |  |  |
|  |  | L statistics | No |  |  |
|  |  | Nearest neighbor correlation | No |  |  |
| Spatial downstream analysis | Spatial clustering | | No | Adjusted rand index (ARI) | [-1, 1] |
|  |  |  | No | Normalized mutual information (NMI) | [0, 1] |
|  | Cell type deconvolution | | No | Root mean square deviation (RMSE) | [0, infinity) |
|  |  |  | No | Jensen-Shannon divergence (JSD) | [0, 1] |
|  | Spatially variable genes (SVG) identification | | No | Precision | [0, 1] |
|  |  |  | No | Recall | [0, 1] |
|  | Spatial cross-correlation | | No | Cosine similarity | [-1, 1] |
|  |  |  | No | Mental statistics | [-1, 1] |
| Scalability | 200 x 200  3k x 500  5k x 1k | | No | time | [0, infinity) |
|  |  |  | No | memory | [0, infinity) |

### 
